# Deciphering drought-response in wheat (*Triticum aestivum*): physiological, biochemical, and transcriptomic insights into tolerant and sensitive cultivars under dehydration shock

**DOI:** 10.3389/fpls.2025.1649378

**Published:** 2025-10-27

**Authors:** Birsen Cevher-Keskin, Yasemin Yıldızhan, A. Hediye Sekmen, Rumeysa Fayetorbay, Osman Uğur Sezerman, Buğra Özer, Selma Onarıcı, İsmail Türkan, Mahmut Tör

**Affiliations:** ^1^ Plant Molecular Biology and Genetics Laboratory, Life Sciences, Marmara Research Centre, The Scientific and Technological Research Council of Türkiye (TUBITAK), Kocaeli, Türkiye; ^2^ Department of Biology, Faculty of Science, Ege University, İzmir, Türkiye; ^3^ Department of Biostatistics and Medical Informatics, Faculty of Medicine, Acıbadem University, Istanbul, Türkiye; ^4^ Molecular Biology, Genetics & Bioengineering, Faculty of Engineering and Natural Sciences, Sabancı University, Istanbul, Türkiye; ^5^ Medical Biology, Faculty of Medicine, Yozgat University, Yozgat, Türkiye; ^6^ Department of Plant & Soil Sciences, Faculty of Agricultural Sciences and Technologies, Yaşar University, İzmir, Türkiye; ^7^ Department of Biological Sciences, School of Science and the Environment, University of Worcester, Worcester, United Kingdom

**Keywords:** drought stress, *Triticum aestivum* L., RNAseq, metal ion binding, ABA signalling, shock- dehydration, antioxidant enzymes

## Abstract

**Introduction:**

Wheat (*Triticum aestivum L.*) is a major staple crop, but its productivity is severely threatened by drought, especially during reproductive stages when yield and quality are most vulnerable. Climate change and water overexploitation intensify this challenge, with yield losses of up to 80% in arid regions and projected global production declines of ~29%. Drought tolerance is a complex trait involving physiological, biochemical, and molecular mechanisms, including stomatal regulation, osmolyte accumulation, and activation of stress-responsive genes. Advances in transcriptomics, functional genomics, and genome editing have identified key regulators (DREB, ERF, SnRK2), antioxidant enzymes, and ABA signalling components as targets for improving drought resilience. Developing drought-tolerant wheat varieties is therefore a priority for food security.

**Materials and Methods:**

This study investigates transcriptomic responses in root and leaf tissues of three wheat cultivars, Atay 85 (drought-sensitive), Gerek 79 and Müfitbey (drought-tolerant), subjected to 4- and 8-hour shock-dehydration stress. Before RNAseq analysis, biochemical assays were conducted to assess oxidative damage (TBARS) and antioxidant enzyme activities under shock-dehydration stress for three different cultivars. Differential gene expression analysis was performed, and several highly differentially expressed genesincluding TaZFP36, TaMC5, TaGI, TaGLP9-1, and TaFer were selected to validate RNAseq data in both root and leaf tissues of tolerant and sensitive cultivars.

**Results:**

Transcriptomic analysis revealed distinct metabolic strategies for drought adaptation. Photosynthesis-related processes, including Photosystem I and II, were broadly downregulated, while extracellular and membrane-associated components were upregulated, reflecting a shift toward stress defence mechanisms. Cultivar-specific responses highlighted diverse adaptation strategies: Atay 85 exhibited severe metabolic suppression and ATP depletion, making it highly vulnerable to drought. Gerek 79 conserved energy by suppressing photosynthesis while enhancing osmoprotective sugar metabolism and reinforcing structural integrity through lignin and flavonoid biosynthesis. Müfitbey demonstrated the most robust drought tolerance by integrating metabolic dormancy, hormonal signalling, and antioxidant defence, characterized by stable CAT activity and elevated SOD activity, which mitigated oxidative damage and preserved photosynthetic stability. Root tissues prioritized metabolic adjustments for oxidative stress reduction and developmental adaptation, while leaf tissues focused on maintaining photosynthesis and limiting protein damage. Functional enrichment analysis indicated significant upregulation of stress-related pathways, including ABA-mediated signalling, protein binding, and cellular metabolic processes in tolerant cultivars.

**Discussion:**

This study advances our knowledge of the complex molecular and biochemical responses of wheat with differing tolerance levels, highlighting both key candidate genes and antioxidant defence mechanisms as central to cultivar-specific adaptation strategies. The distinct metabolic strategies observed emphasize the importance of tailored molecular mechanisms in drought tolerance, which can guide future breeding programs aimed at improving wheat resilience under water-limited conditions.

## 1 Introduction

Bread wheat, *Triticum aestivum* L. is one of the staple crops in many countries. According to the Food and Agriculture Organization of the United Nations (FAO), global wheat production was estimated at 766.5 million tons in 2020 (www.fao.org) (Food and Agriculture Organization of the United Nations, 2024), and the requirement for wheat is expected to rise by 60% by 2050. Drought is a major threat to wheat, reducing grain yield, kernel weight, and end-use quality, particularly during heading and grain filling (Zampieri et al., 2017; Bagci et al., 2007). The problem is acute in arid regions such as central and eastern Anatolia, Turkey. Yield losses can reach up to 80% in some years, especially in central Turkey (Turkish Statistical Institute 2024), where groundwater resources have nearly been depleted due to the excessive use for irrigation, further exacerbating the problem. Flowering and grain development stages are the most drought-sensitive growth stages, with stress at these points reducing both yield and grain protein quality. In addition, climate change is projected to further reduce wheat production by up to 29% (Manickavelu et al., 2012). These predictions clearly show that the improvement of drought tolerance in wheat is of great significance for the global food security in the near future. Genetic studies and new approaches to improve wheat productivity under drought conditions is an urgent priority.

Drought stress tolerance is a complex trait that involves physiological, biochemical, and molecular processes. Adaptation strategies in drought-tolerant plants include reducing water loss through increased stomatal resistance, enhancing water uptake via larger and deeper root systems, and accumulating osmolytes such as proline, glycine betaine, mannitol, sorbitol, trehalose, and glutamate (Mahajan and Tuteja, 2005).

Plant responses to drought stress start with the stimulation of signal transduction cascades. The activation of several transcription factors and regulators initiates the induction of several molecular and cellular mechanisms. Depending on the genetic background, the response to drought stress varies considerably. For instance, Iqbal et al. (2019) reported distinct water stress responses in two genetically different soybean genotypes, demonstrating the diversity of drought adaptation strategies. Transcriptomic, proteomic, and genetic manipulation studies have identified several key genes and enzymes potentially involved in drought tolerance. These include transcription factors such as Dehydration-Responsive Element Binding Factor 1 (DREB1B) and Ethylene Responsive Factor 3 (ERF3); signalling proteins like SNF1-Associated Protein Kinase 2 (SnRK2); enzymes involved in ABA biosynthesis such as Zeaxanthin Epoxidase (ZEP) and 9-cis-Epoxycarotenoid Dioxygenase (NCED); plasma membrane intrinsic proteins (PIPs); and a suite of antioxidant enzymes including peroxidase (POD), glutathione reductase (GR), catalase (CAT), superoxide dismutase (SOD), ascorbate peroxidase (APX), dehydroascorbate reductase (DAR), and guaiacol peroxidase (GPOX) (Alzahrani et al., 2018; Verma et al., 2020). Improved water-use efficiency has been achieved by the knockout of TaERF3 under drought stress (Rong et al., 2014). Overexpression of the DREB1A gene from Arabidopsis has led to enhanced drought tolerance in wheat by improving osmoprotectant accumulation and reducing water loss (Qain-Ali et al., 2021). In Arabidopsis, activation of drought-regulated genes by AtSnRK2.8 involved in ABA signalling, drought resistance, and plant growth demonstrated a key stress regulatory network that improves drought resistance (Umezawa et al., 2004). Improved plant growth and abiotic stress response were observed in rice with the presence of sub-class I and III SnRK2 family members (Kulik et al., 2011).

Microarray and RNA-seq analyses have revealed numerous genes associated with abiotic stress responses, particularly drought stress, across diverse plant species (Liang et al., 2017; Li et al., 2020; Abdel-Ghany et al., 2020). In T. aestivum genes related to photosystem components, carbohydrate metabolism, antioxidant enzymes, and the tricarboxylic acid cycle have been identified as key contributors to drought tolerance (Peremarti et al., 2014). During reproductive stages, more than 300 differentially expressed genes (DEGs) associated with photosynthesis, stomatal regulation, and floral development have been reported under drought stress (Ma et al., 2017). Key transcription factors, including WRKY, ERF, NAC, bHLH, bZIP, HD-ZIP, as well as dehydrins, heat shock proteins, proteinase inhibitors, and glutathione transferases, constitute the main DEGs responsive to drought (Kulkarni et al., 2017). Genes in the antioxidant defence system such as Fe/Mn SOD, PER1, PER22, SPC4, CAT2, APX1, APX7, GSTU6, GST4, GOR, GRXC1, and GRXC15 are upregulated in response to drought stress, mediated in part by phytohormone strigolactones (SLs) (Song et al., 2023). Additionally, glutathione S-transferase (GST), RAB, rubisco, helicase, and vacuolar acid invertase genes have been linked to drought tolerance in wheat (Nezhadahmadi et al., 2013).

In Oryza. sativa, Late embryogenesis abundant (LEA) proteins accumulate under drought, salinity, and low temperatures, playing a crucial role in stress adaptation (Xiao et al., 2007). Expression profile analysis determined that most of the GhLEA genes were expressed at a higher rate in drought-resistant cotton varieties than in sensitive ones (Magwanga et al., 2018). Similarly, the accumulation of members of the dehydrin (DHN) family has been linked to stress tolerance involving dehydration in several species, including sunflower (Cellier et al., 1998), cotton (Magwanga et al., 2018), and wheat (Lopez et al., 2003).

Bogard et al. (2021) emphasized that genotypic characteristics related to abiotic stress tolerance should be taken into account in the selection of suitable wheat varieties for breeding in different regions. They developed a marker-based statistical model to predict phenology parameters in wheat and simulated genotype-specific stress avoidance frequencies for frost and heat stress across different locations. The model’s predictions were validated by assessing grain yield performance in a real trial network conducted during low frost and heat risk periods at each location (Bogard et al., 2021). Since the drought stress regulation of some genes has not been completely identified yet, our knowledge of genes involved in drought response is still incomplete.

Recent advances in genome editing, particularly CRISPR/Cas9 technology, have enabled targeted improvement of drought tolerance in plants (Erdoğan et al., 2023). CRISPR/Cas9 has been used to modify key genes in drought-response pathways across various crops. For example, in wheat, TaDREB2 and TaERF3 were edited to improve drought tolerance (Kim et al., 2018). In O. sativa, mutations in SAPK2 and OsERF109 enhanced drought resilience by modulating ABA signalling (Lou et al., 2017). Editing the OsDST gene in O. sativa cultivar MTU1010 improved drought and salt tolerance, promoting leaf retention under stress (Santosh Kumar et al., 2020). In tomato, knock-out of SlMAPK3 increased drought tolerance, marked by elevated malondialdehyde, proline, and H_2_O_2_ levels, while knock-out of SlNPR1 reduced drought resistance (Li et al., 2019). In wheat, silencing Sal1 enhanced drought tolerance (Abdallah et al., 2022). Similarly, in maize, editing ARGOS8 and ZmWRKY40 improved drought resilience (Shi et al., 2017; Wang C. T. et al., 2018).

This study was aimed to discover genes that are responsive to drought stress in bread wheat (*Triticum aestivum* L.). Through physiological screening, we identified wheat cultivars displaying varying levels of sensitivity and tolerance to drought. Leveraging RNA-Seq technology, we examined expression profiles of drought-responsive genes within the leaves and roots of three distinct wheat cultivars following exposure to shock dehydration stress conditions. Our investigation unveiled a considerable number of genes exhibiting either elevated or decreased levels of expression in both drought-tolerant and sensitive bread wheat cultivars. Subsequently, selected DEGs were validated using quantitative real-time polymerase chain reaction (qRT-PCR). The insights gained from this research have the potential to inform the development of drought-tolerant wheat varieties, employing diverse methodologies, including genome-editing techniques.

## 2 Materials and methods

### 2.1 Plant materials

Seeds of twelve T. aestivum cultivars, classified as drought-tolerant or drought-sensitive, were obtained from the General Directorate of Agricultural Research and Policies (TAGEM), Turkey (**Supplementary Table S1**). The seeds were surface sterilized (5 min with 70% EtOH and 5 min with 5% hypochlorite) and pre-germinated in Petri dishes for 10 days on moist filter paper at 4°C in the dark. Uniform seedlings were transplanted into 1.5 L plastic pots filled with a turf: soil: sand mixture (3:3:1) and grown in a controlled environment chamber at 18–20°C, 60–70% relative humidity. For each cultivar, three pots were assigned to the control and three to the drought-stress treatment.

#### 2.1.1 Drought stress treatment and selection of cultivars for further investigations

Progressive drought stress was initiated three weeks after transferring the seedlings to the pots and carried out by withholding water from the stress treated pots. A regular watering regime was carried out for the control plants every day. Soil Water Content (SWC) measurements were taken during the stress. At the end of the tenth day of drought treatment, Relative Water Content (RWC) measurements were calculated for each cultivar as described (Barrs and Weatherley, 1962). All plants were harvested at the end of the 10^th^ day of drought treatment. Harvested tissues were directly frozen in liquid nitrogen and stored at -80°C till use. For each pot, three different measurements were taken in the afternoon for every day. Based on the physiological data (RWC, SWC), from the three biological replicates of each cultivar, drought-sensitive and drought-tolerant bread wheat cultivars were identified. After drought treatment, physiological resilience and Relative Water Content (RWC) levels were assessed. RWC experiment showed Gerek 79, Müfitbey, Altay, and Harmankaya-99 highly drought-tolerant cultivars maintain high RWC levels even under drought stress, suggesting better water retention and drought tolerance (**Supplementary Figure S1**).

Our findings showed that Gerek 79 and Müfitbey maintained higher relative water content (RWC) and exhibited strong antioxidant enzyme responses under drought stress, indicating resilience, whereas Atay 85 displayed significant water loss and lower antioxidant activity, suggesting drought sensitivity These three Turkish winter bread wheat (*Triticum aestivum* L.) cultivars, officially registered by the Ministry of Agriculture and Forestry (Geçitkuşağı Agricultural Research Institute, Eskişehir), represent contrasting drought responses. Müfitbey is classified as drought-tolerant with stable photosynthetic activity and canopy cooling under post-anthesis stress, while Gerek 79 serves as a widely recognized drought-tolerant check adapted to rainfed conditions. In contrast, Atay 85 is generally considered drought-sensitive and is often used as a susceptible control in multi-environment trials (Tarım Orman, 2025a; NBC Agriculture). These physiological and biochemical differences made them ideal candidates for RNA-seq analysis, with Gerek 79 and Müfitbey selected as drought-tolerant and Atay 85 as drought-sensitive for transcriptomic profiling.

#### 2.1.2 Soil water content

The Time Domain Reflectometry (TDR) Soil Moisture System (Spectrum Technologies, Illinois) was used for the estimation of the mean soil moisture. During the progressive drought stress application, soil moisture ratios were measured in pots of drought and control plant samples for each of the 12 cultivars every day.

#### 2.1.3 The relative water content

At the end of 10 days of drought stress, leaf tissues (the third youngest leaf) were collected for RWC measurements. RWC quantifications were performed as described by Barr and Weatherley (1962). Fresh leaves (0.5 g) were cut into 1-cm- long fragments and weighed for their fresh weight (FW), then saturated in water for 8 hours at 4°C and weighed for their turgid weight (TW). Subsequently, the samples were dried in an oven at 80°C for 24 hours, and the dry weight (DW) was measured. The RWC was calculated by using the formula (FW-DW)/(TW-DW) X 100% (**Supplementary Figure S2**).

#### 2.1.4 Shock dehydration stress

**Figure 1:**
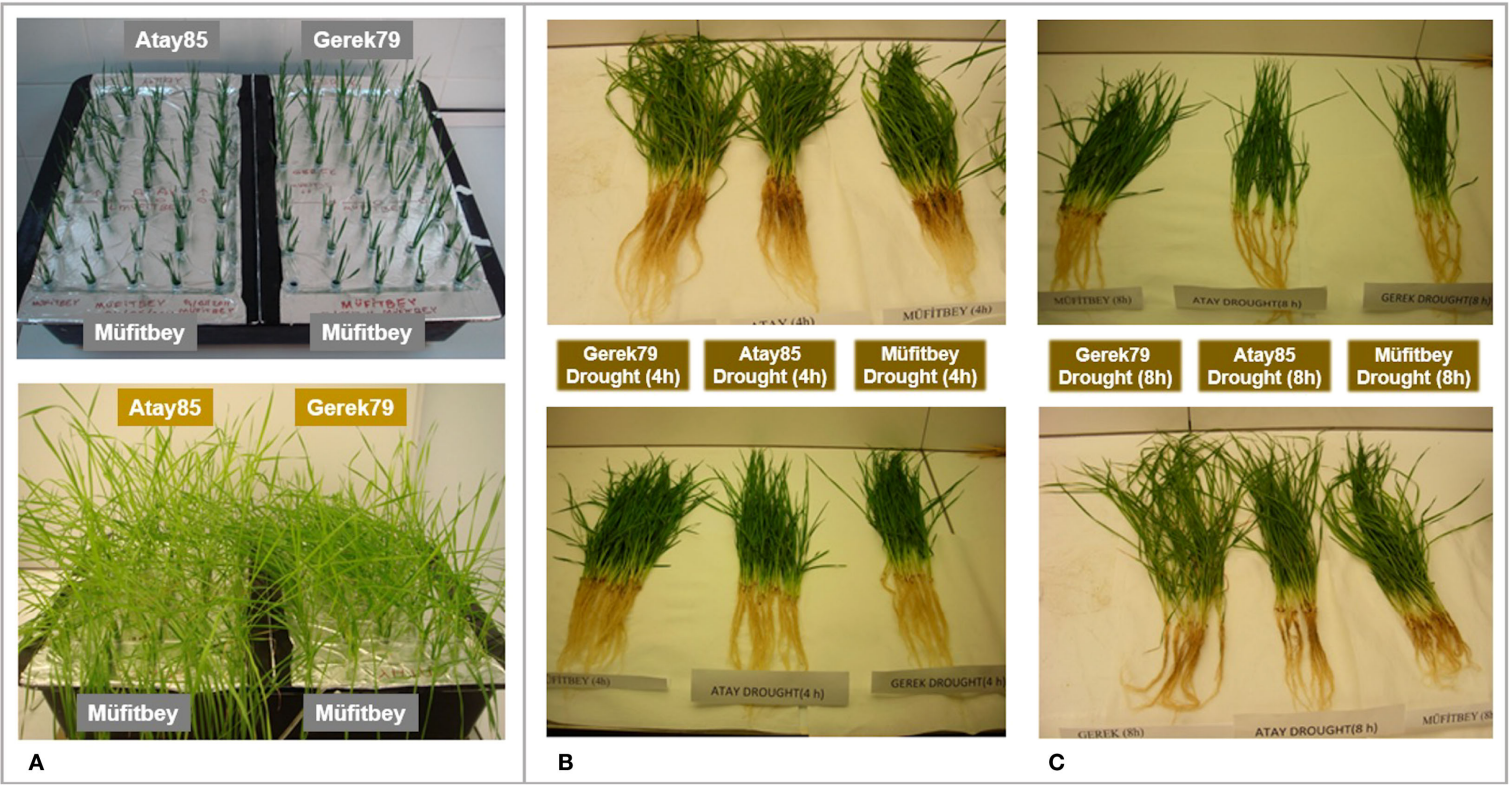
Shock dehydration stress induction in Gerek 79, Atay 85, and Müfitbey cultivars. **(A)** Seedlings at a similar developmental stage were transferred to continuously aerated ½ Hoagland’s solution, renewed every three days, and grown under controlled conditions (16 h photoperiod, 22/18°C temperature, and 60% relative humidity). **(B)** 4 hours and **(C)** 8 hours after removal from hydroponic culture.

To identify more rapid changes in drought related gene expression, a shock dehydration experiment was performed on wheat cultivars differing in drought tolerance: the drought-tolerant cultivars (Müfitbey and Gerek 79) and the sensitive cultivar (Atay 85), as described by Ergen et al. (2009) with some modifications. Seeds were surface sterilized in 70% EtOH for 5 minutes and in 30% sodium hypochlorite for 10 minutes. Subsequently, seeds were rinsed six times with sterile distilled water for 2 minutes and pre-germinated in Petri dishes for 10 days at 4°C in the dark. Following germination, seedlings were transferred to 10 L plastic pots containing moistened perlite for initial growth. Seedlings of a similar developmental stage were then transferred to a continuously aerated ½ Hoagland’s solution renewed every 3 days, and grown under controlled conditions (16h photoperiod, temperature 22/18°C and relative humidity 60%). For shock dehydration stress, seedlings of each cultivar were removed from hydroponic culture and placed on the laboratory bench at room temperature for 4 or 8 hours. Control plants remained in hydroponic culture and were harvested at the same time points without exposure to dehydration stress (**Figure 1**). Leaf and root tissues from three biological replicates of each cultivar were analyzed under four conditions (4 hours drought, 4 hours control, 8 hours drought, and 8 hours control), resulting in a total of 72 samples (3 genotypes × 4 conditions × 3 replicates × 2 tissues).

### 2.2 Determination of lipid peroxidation

Lipid peroxidation was quantified by measuring thiobarbituric acid reactive substances (TBARS), a widely used indicator of oxidative membrane damage under stress conditions. TBARS levels were determined following the method of Madhava Rao and Sresty (2000), using an extinction coefficient of 155 mM^-^¹ cm^-^¹ for calculation.

### 2.3 Enzyme extraction and protein determination

To further understand the antioxidant defense strategies, the activities of key ROS-scavenging enzymes were assessed. All enzyme extractions were carried out at 4°C. Fresh tissue samples (0.5 g) were ground in liquid nitrogen and homogenized in 1.5 ml of 50 mM Tris-HCl buffer (pH 7.8) containing 0.1 mM EDTA, 0.2% (w/v) Triton X-100, 1 mM phenylmethylsulfonyl fluoride (PMSF), and 2% (w/v) polyvinylpyrrolidone (PVP). For ascorbate peroxidase (APX) extraction, 5 mM ascorbate was added to the homogenization buffer. The homogenates were centrifuged at 14,000 × g for 30 min, and the resulting supernatants were used for protein quantification and enzyme assays. Total soluble protein content was determined using the Bradford method with bovine serum albumin as a standard. All spectrophotometric measurements were performed using a Shimadzu UV-1600 spectrophotometer.

#### 2.3.1 Antioxidant enzyme activity assays

Superoxide dismutase (SOD; EC 1.15.1.1): Assayed according to Beauchamp and Fridovich (1971) by monitoring the inhibition of nitroblue tetrazolium (NBT) photoreduction at 560 nm. One unit of SOD activity was defined as the amount of enzyme required to inhibit NBT reduction by 50%.

Catalase (CAT; EC 1.11.1.6): Measured as the decline in H_2_O_2_ absorbance at 240 nm (Bergmeyer, 1970). One unit corresponded to the decomposition of 1 mmol H_2_O_2_ min^-^¹.

Peroxidase (POX; EC 1.11.1.7): Assayed by monitoring guaiacol oxidation at 465 nm (Herzog and Fahimi, 1973). One unit was defined as the decomposition of 1 mmol H_2_O_2_ min^-^¹.

Ascorbate Peroxidase (APX; EC 1.11.1.11): Determined by the decrease in ascorbate absorbance at 290 nm (Nakano and Asada, 1981), using an extinction coefficient of 2.8 mM^-^¹ cm^-^¹. One unit was defined as the oxidation of 1 mmol ascorbate min^-^¹.

### 2.4 Isolation of total RNA

Total RNA isolation was performed from leaf and root tissues of 4- and 8-hours droughts stressed and control groups using the RNeasy Plant Mini kit (Qiagen, Hilden, Germany) according to the manufacturers’ instructions. Approximately 100 mg of tissue was ground into a fine powder using a pre-chilled mortar and pestle with liquid nitrogen. The powdered tissue was transferred to lysis buffer containing guanidine thiocyanate to inactivate RNases, followed by homogenization through the kit-provided shredder column to remove cellular debris. RNase-free DNaseI (Roche Applied Science GmbH, Germany) digestion and purification were carried out for the elimination of the genomic DNA from total RNA as described Cevher-Keskin et al. (2019). RNA concentration and purity were initially determined using a NanoDrop ND-1000 spectrophotometer (Thermo Fisher Scientific, USA), ensuring A260/A280 ratios between 1.9 and 2.1 and A260/A230 ratios above 2.0. RNA integrity was further assessed with an Agilent 2100 Bioanalyzer (Agilent Technologies, USA), and only samples with an RNA Integrity Number (RIN) ≥ 8.0 were selected for downstream RNA-seq analysis. All samples were stored at –80 °C until library preparation.

### 2.5 RNA sequencing

Leaf and root tissues from three biological replicates of each cultivar were analyzed under four conditions (4-hours drought, 4-hours control; 8-hours drought, and 8-hours control), resulting in a total of 72 samples (3 genotypes × 4 conditions × 3 replicates × 2 tissues). The RNAseq library for each sample was prepared with a 1250 ng of total RNA using the TruSeq RNA Sample Preparation kit (Illumina) according to the manufacturer’s instructions. Paired-end sequencing was performed with a current next generation sequencing instrument, HiSeq2000 (Illumina, user guide; Part# 15011190 Rev. H) using TruSeq SBS Kit v3 (cBot-HS) (Illumina, user guide; Part#15023333 Rev. B). The prepared libraries were enriched using 15 cycles of PCR and purified by the QIAquick PCR purification kit (Qiagen). The Agilent 2100 Bioanalyzer was used to control the size and purity of the samples using the Agilent High Sensitivity DNA Kit. A total of 12 indexes were prepared for 72 samples and run-on Illumina HiSeq 2000 for 6 lanes (Pingault et al., 2015). The enriched libraries were diluted with the elution buffer to a final concentration of 10 nM. Sequencing was performed on each library to generate 100-bp PE reads for transcriptome sequencing on an Illumina High-Seq 2000 platform.

### 2.6 Differential gene expression analysis

The quality control was performed for the Illumina paired-end sequencing files of each sample. FastQC Software” was used for the detection of faulty sequences (Andrews, 2022). RNA-seq data were trimmed using the Fastx Toolkit (http://hannonlab.cshl.edu/fastx_toolkit, Hannon Lab, 2022). After quality control, *de novo* assembly was carried out from a total of 311 GB of transcript data. The assembly was performed as recommended by Duan et al. (2012). The resultant data were evaluated using the software “Trinity Assembly”, which combines three independent software modules (Inchworm, Chrysalis and Butterfly) and 323 Mbs of FASTA files were obtained. To remove the expected redundancy in this assembly file, “the cd-hit-est tool” to place the contigs into clusters was applied, so that a sequence is not represented more than once in our reference assembly. Subsequently, the RNA-seq data were mapped to our *de novo* reference genome using Bowtie (https://bowtie-bio.sourceforge.net/index.shtml, 2022; Langmead et al., 2022). The resulting mapped reads were evaluated by using the RSEM tool to obtain Fragments per Kilobase of transcript per Million mapped reads (FPKM) data. FPKM files belonging to each sample were subjected to pairwise comparison using the edgeR differential expression tool, which is included in the R-Bioconductor package (Robinson et al., 2010). Through differential expression analysis, we pooled replicates belonging to each condition into a single file by averaging the counting information corresponding to each gene. As a result, comparisons between different conditions were carried out and differentially expressed transcripts were obtained. However, some transcripts were not informative, as they were not annotated due to a lack of well-annotated reference genome. In this case, the Trinotate annotation tool (https://rnabio.org/module-07-trinotate/0007/02/01/Trinotate) was used which uses various well referenced methods for functional annotation including homology search for known sequence data (NCBI-BLAST), protein domain identification (HMMER/PFAM), protein signal prediction (singalP/tmHMM), and comparison to currently curated annotation databases (EMBL Uniprot eggNOG/GO Pathways databases) have been applied. To account for multiple hypothesis testing, we first calculated adjusted p-values using the Benjamini-Hochberg procedure, which controls the expected proportion of false positives (Benjamini and Hochberg, 1995; Benjamini and Yekutieli, 2001). These adjusted p-values were recommended as the primary statistic for interpreting significance. Additionally, multiple testing corrections were performed using the False Discovery Rate (FDR) method to further control type I error, applying a stringent threshold of FDR ≤ 0.05 for the identification of DEGs and enriched functional terms. In addition to this statistical threshold, we assessed the biological relevance of the expression change using the log_2_ Fold Change (log_2_FC), which quantified both the direction (up- or down-regulation) and the magnitude of the transcriptional difference. A log_2_ fold change threshold was applied to filter genes, ensuring that only those with biologically meaningful shifts were retained for downstream analysis. Genes exceeding the defined cut-off, either positively or negatively, were considered significant and included in the final dataset. The colour intensity, based on the adjusted logarithmic scale of FC values, demonstrates the level of significance of each term. If there was no log2FC score for the corresponding enriched term, this was depicted as white in the heatmap. Pathway enrichment analysis is performed by using WebGestaltR. The terms belong to KEGG and WikiPathways. Adjusted values indicate adjusted p-values. Negative logarithm base 10 was applied to the p-values of pathway terminologies.

### 2.7 Primer design for qRT-PCR

Primers were designed for the selected genes using FastPCR and Primer 3 programs. The quality of the primers was validated by BLASTN queries against the entire wheat EST unigene set. The primers, wherever possible, were designed spanning an intron or intron-intron junctions to detect any genomic DNA contamination. All the primers were adjusted to 100–140 bp amplicon size and 55°C annealing temperature and controlled by conventional PCR by housekeeping genes (β actin, EF-1 and EF2 primers).

### 2.8 cDNA synthesis and qRT-PCR

First-strand cDNA was synthesized from 1 μg of total RNA in a 20 μl reaction using MMLV reverse transcriptase (Roche High Fidelity cDNA Synthesis Kit) following the manufacturer’s instructions. cDNA quality was verified by conventional PCR using housekeeping gene primers (β-actin, EF1, EF2). Differential expression analysis was performed with SYBR Green Mix (Roche FastStart Universal SYBR Green Master) and gene-specific primers (**Supplementary Table S5**) on an iQ5 System (Bio-Rad, Hercules, USA) as described by Cevher-Keskin et al. (2011). Each reaction was run in triplicate to ensure accuracy. Relative transcript abundance was normalized to housekeeping genes (EF-α1 and EF-α2), and fold changes were calculated using the comparative CT (ΔΔCq) method (Schmittgen et al., 2000). Error bars represent standard deviation across three technical replicates.

### 2.9 Statistical analysis

Data from three independent biological replicates were analyzed using one-way analysis of variance (ANOVA), or Student’s t-test, as appropriate, to evaluate the effects of treatments on the measured parameters. When significant differences were observed, Tukey’s Honest Significant Difference (HSD) *post hoc* test was applied for pairwise comparisons. Statistical significance was set at p < 0.05 (*, significant) and p < 0.01 (**, highly significant); results with p ≥ 0.05 were considered not significant (ns). All statistical analyses were performed using GraphPad Prism (v. 10.5.0 for Mac; GraphPad Software). Bar graphs for enzyme analysis were generated using Microsoft Excel (Microsoft Corporation, Redmond, WA, USA).

## 3 Results

### 3.1 Physiological screen of wheat cultivars reveals drought-tolerant and sensitive genotypes

In the present study, 12 bread wheat (*Triticum aestivum* L.) cultivars with diverse genetic backgrounds were initially evaluated to determine the most promising drought stress tolerant and sensitive cultivars under progressive drought stress (**Supplementary Figure S2**).

Initially, we conducted a physiological screen. Soil water content (SWC) was monitored throughout the drought stress period. In control plants, SWC ranged from 35% to 45%, depending on the cultivar. Under drought conditions, SWC generally declined to 8–22%. Some cultivars, such as Sultan, Kırgız, and Gerek, showed a larger SWC difference between control and drought treatments, suggesting faster water depletion or higher water loss. In contrast, Müfitbey and Harmankaya-99 exhibited smaller differences, indicating slower water uptake or more efficient water conservation. Relative water content (RWC) also decreased during the 10-day drought treatment, with Atay 85 showing the most pronounced reduction, dropping below 70% (**Supplementary Figure S2**). Conversely, Gerek 79, Müfitbey, Altay, and Harmankaya-99 maintained higher RWC levels under stress, reflecting better water retention and drought tolerance.

To contextualize these findings, we reviewed agronomic and historical performance data from the Ministry of Agriculture (Geçitkuşağı Agricultural Research Institute, Eskişehir, Turkey). Müfitbey is a drought-tolerant winter cultivar, maintaining photosynthetic function, canopy cooling, and stable PSII efficiency (Fv/Fm) under stress (Dirik and Sakin, 2024; Tarım Orman, 2025a). Gerek 79, widely used as a tolerant check, shows broad adaptation and resilience to combined drought and heat stresses (Tarım Orman, 2025b). In contrast, Atay 85 is drought-sensitive, with sharp declines in RWC, SPAD, and Fv/Fm under water deficit (NBC Agriculture). Atay 85 performs best under irrigation, whereas Gerek 79 thrives in drought-prone, cold regions, and Müfitbey combines winter hardiness with drought tolerance. Based on these contrasting traits, we selected Gerek 79 (tolerant), Müfitbey (highly tolerant), and Atay 85 (sensitive) for further analysis.

To capture robust transcriptional responses, we applied a shock dehydration treatment, as acute stress elicits stronger gene expression changes than progressive drought. Seedlings at a similar developmental stage were grown in aerated ½ Hoagland’s solution (renewed every three days) under controlled conditions (16 h photoperiod, 22/18 °C, 60% RH) (**Figure 1A**). They were then removed and subjected to 4 and 8 hours of shock dehydration (**Figure 1B, C**). Leaf and root tissues were immediately frozen in liquid nitrogen, stored at −80 °C, and used for RNA-seq.

### 3.2 Drought-tolerant and sensitive wheat cultivars exhibit contrasting antioxidant responses

Lipid peroxidation (TBARS assay) used as an indicator of oxidative membrane damage under drought stress. To elucidate antioxidant defense strategies, we analyzed the activities of key ROS-scavenging enzymes: superoxide dismutase (SOD), catalase (CAT), peroxidase (POX), and ascorbate peroxidase (APX). SOD catalyzes the dismutation of superoxide radicals, protecting photosynthetic pigments and stabilizing PSII under stress (Rao et al., 2025). CAT complements this by decomposing hydrogen peroxide into water and oxygen, maintaining redox balance (Calderón et al., 2018). Together with POX and APX, these enzymes form a coordinated antioxidant network mitigating drought-induced oxidative stress.

**Figure 2:**
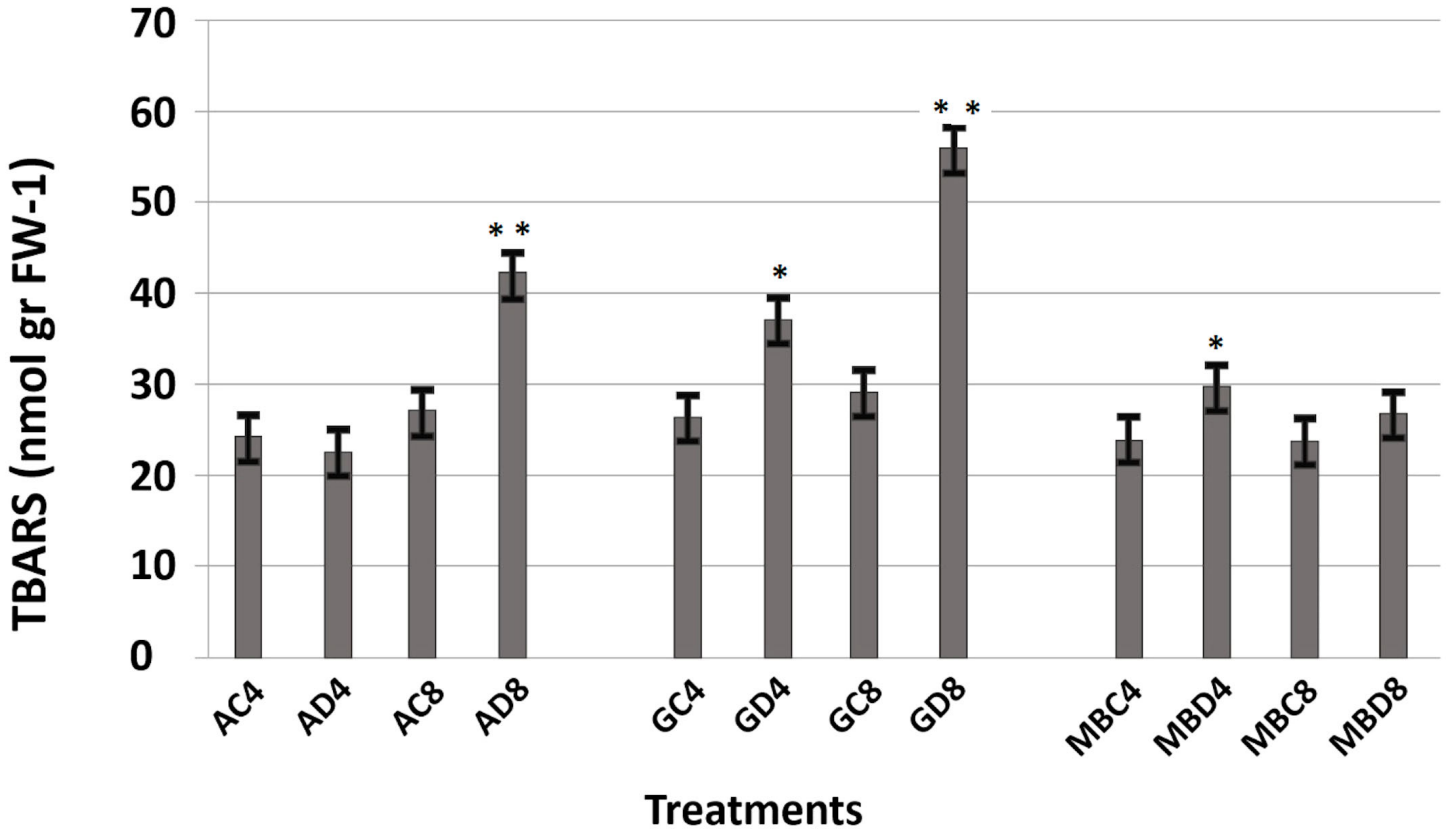
The TBARS contents in the leaves of the three wheat genotypes showed significant variation in response to drought stress and exposure time. Drought stress induced genotype-dependent increases in TBARS contents. The highest accumulation was observed in Gerek 79 at 8 h (GD8), followed by Atay 85 (AD8), while Müfitbey showed comparatively stable and lower levels, indicating greater tolerance to oxidative damage. Values represent means ± SE (n = 3). Statistical significance was assessed using one-way analysis of variance (ANOVA) to evaluate the effects of treatments on the measured parameters. When significant differences were observed, Tukey’s Honest Significant Difference (HSD) *post hoc* test was applied for pairwise comparisons. Statistical significance was set at p < 0.05 (*significant) and p < 0.01 (**highly significant); results with p ≥ 0.05 were considered not significant.

**Figure 3:**
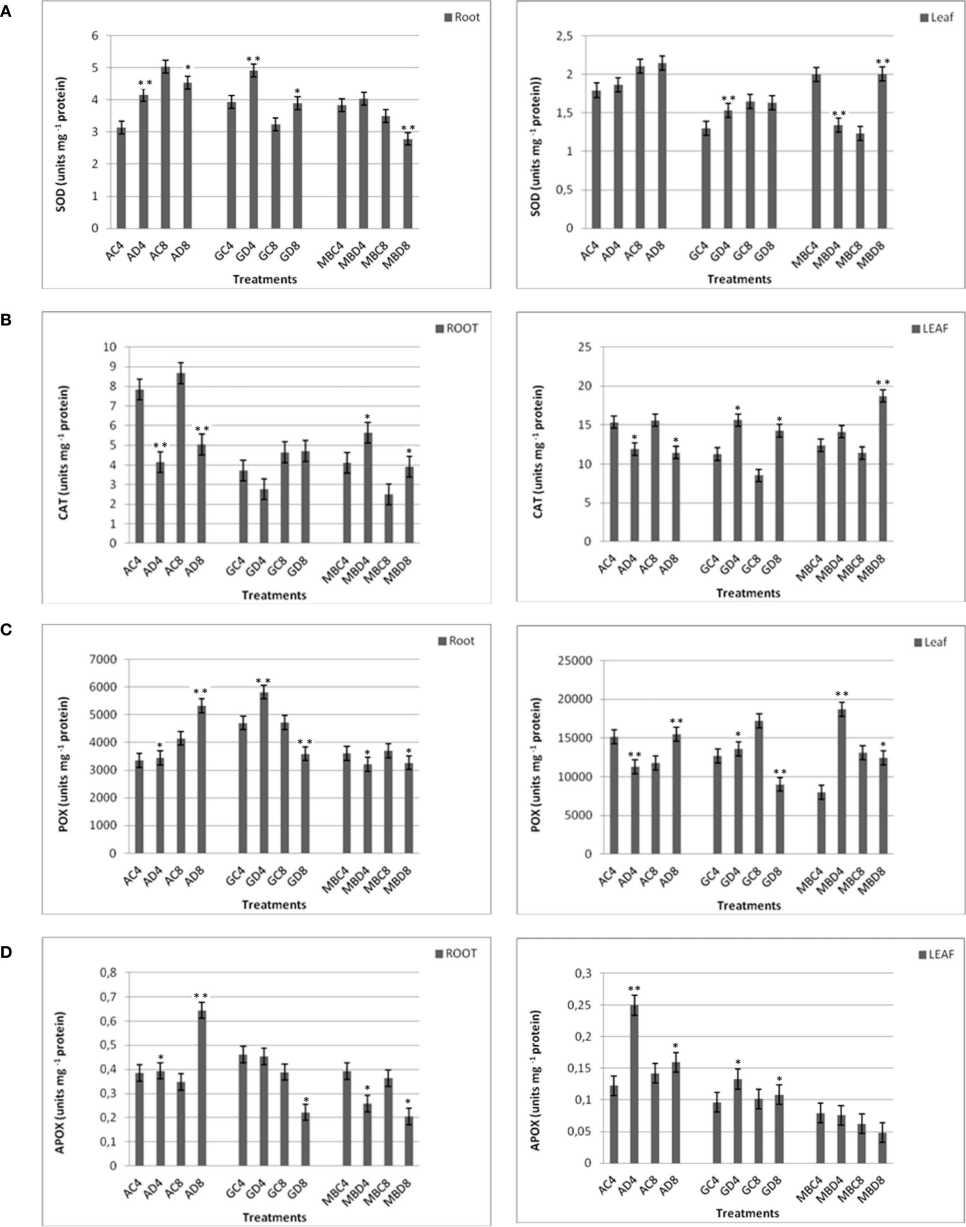
Antioxidant enzyme activities in roots and leaves of wheat genotypes under drought stress. **(A)** Superoxide dismutase (SOD), **(B)** Catalase (CAT), **(C)** Peroxidase (POX), and **(D)** Ascorbate peroxidase (APX) activities were measured in roots (left panels) and leaves (right panels) of three wheat genotypes (Atay 85, Gerek 79, Müfitbey) subjected to drought stress for 4 h (D4) and 8 h (D8), along with corresponding controls (C4, C8). Values represent means ± SE (n = 3). Statistical significance was assessed using one-way analysis of variance (ANOVA) to evaluate the effects of treatments on the measured parameters. When significant differences were observed, Tukey’s Honest Significant Difference (HSD) *post hoc* test was applied for pairwise comparisons. Statistical significance was set at p < 0.05 (*significant) and p < 0.01 (**highly significant); results with p ≥ 0.05 were considered not significant.

TBARS levels varied significantly among genotypes and time points (**Figure 2**). Atay 85 showed a sharp increase at 8 hours (AD8), indicating severe oxidative damage. Gerek 79 exhibited the highest TBARS accumulation at GD8, while Müfitbey maintained consistently low levels, suggesting superior oxidative stress tolerance. SOD activity displayed tissue- and genotype-specific patterns (**Figure 3A**). Atay 85 roots showed strong induction at 4 hours and remained high at 8 hours, with leaves following a similar trend. Gerek 79 roots increased at 4 hours, but leaves showed marked suppression under drought. Müfitbey maintained stable SOD activity in both tissues, with only minor fluctuations. CAT responses were highly genotype-dependent (**Figure 3B**). Atay 85 roots exhibited the strongest induction, peaking at AD8 (>9 units mg^-^¹ protein), whereas Gerek 79 roots declined steadily under stress. Müfitbey showed moderate root responses. In leaves, Atay 85 activity decreased under stress, while Gerek 79 and Müfitbey increased, with Müfitbey reaching the highest levels at 8 hours. POX activity highlighted contrasting strategies (**Figure 3C**). Atay 85 roots showed delayed induction, while Gerek 79 roots peaked at 4 h before declining. In leaves, Müfitbey exhibited a rapid and strong POX increase (~135% at 4 h), whereas Atay 85 decreased, suggesting reliance on alternative antioxidant systems. APX responses were also genotype-specific (**Figure 3D**). Atay 85 strongly upregulated APX in both roots and leaves, particularly at 8 h (+84.8%), indicating a robust ascorbate–glutathione cycle. Conversely, Gerek 79 and Müfitbey roots showed significant reductions, and Müfitbey leaves declined at 8 hours, suggesting limited APX contribution under prolonged stress.

### 3.3 Differential gene expression profiles reveal divergence of drought response strategies

**Figure 4:**
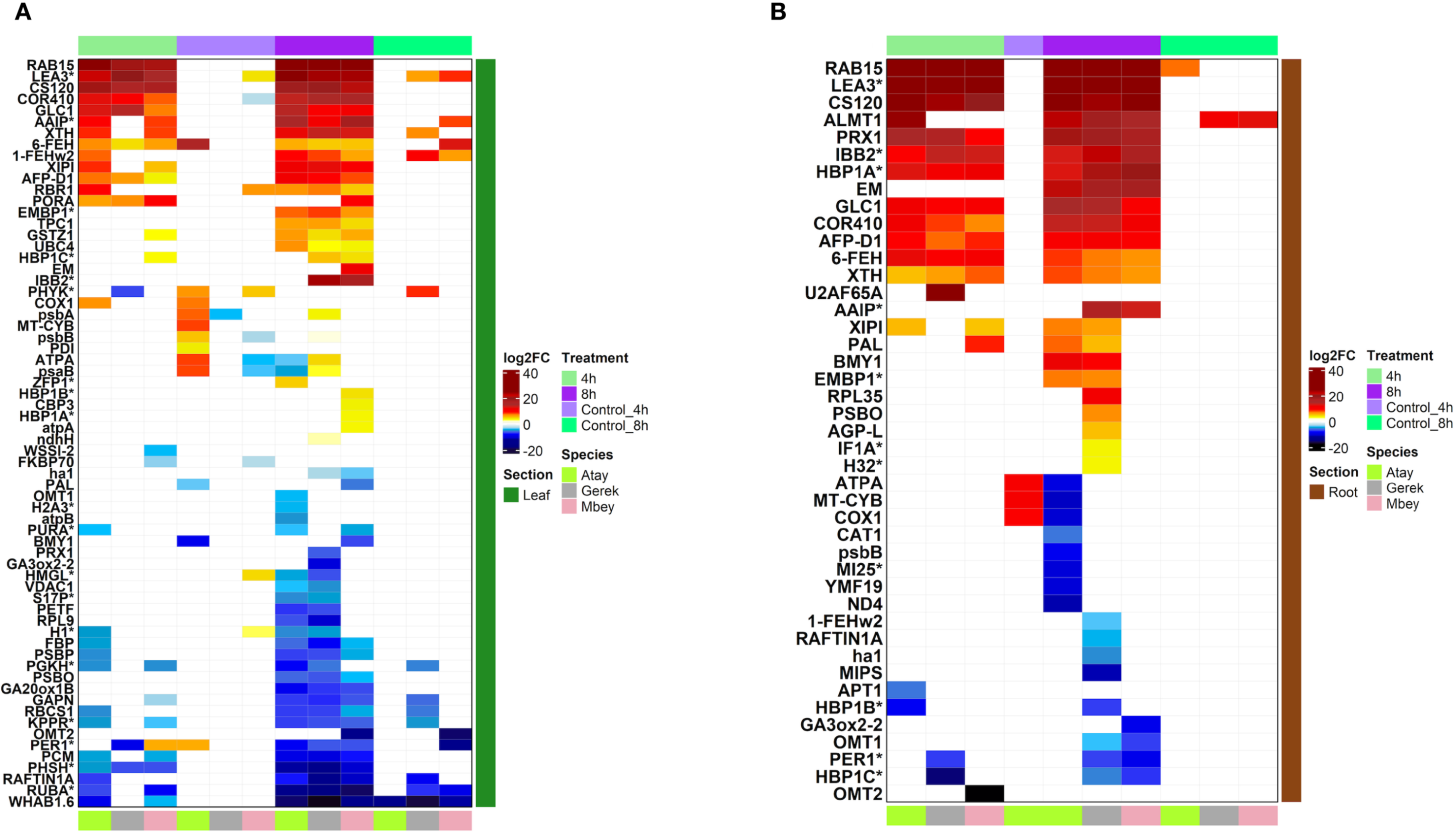
Differentially expressed genes (DEGs) in leaf **(A)** and root **(B)** tissues of drought-tolerant (Gerek 79, Müfitbey) and drought-sensitive (Atay 85) cultivars under 4- and 8-hour drought stress, compared to untreated controls. Red and blue indicate higher and lower expression values, respectively. Cultivars are color-coded as Atay 85 (green), Gerek 79 (grey), and Müfitbey (pink). Significant DEGs were identified at FDR ≤ 0.05. Genes marked with an asterisk (*) represent wheat DEGs as designated in the UniProt database.

**Figure 5:**
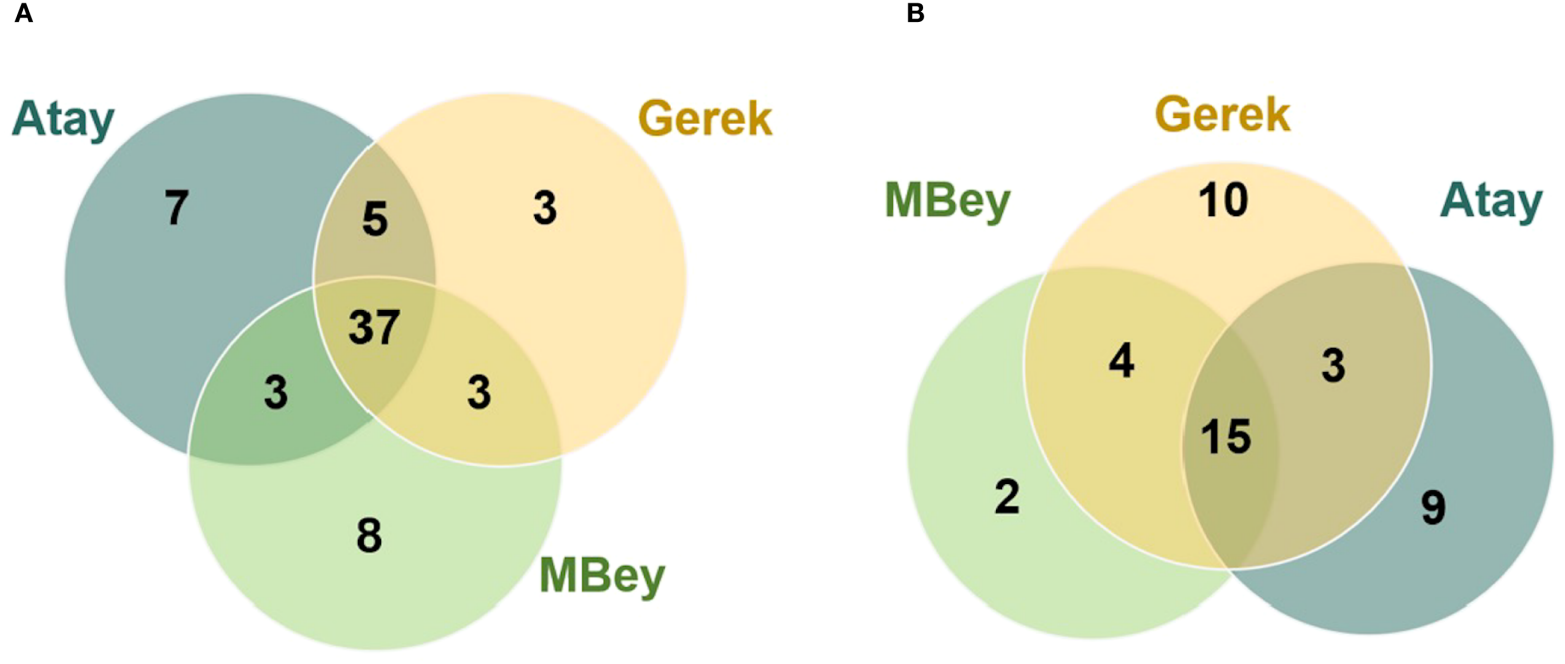
Venn diagrams of differentially expressed genes (DEGs) in leaf **(A)** and root **(B)** tissues. The diagrams display significant DEGs for each cultivar at an FDR threshold of ≤ 0.05. Atay 85 is represented in dark green, Gerek 79 in orange, and Müfitbey in light green.

**Figure 6:**
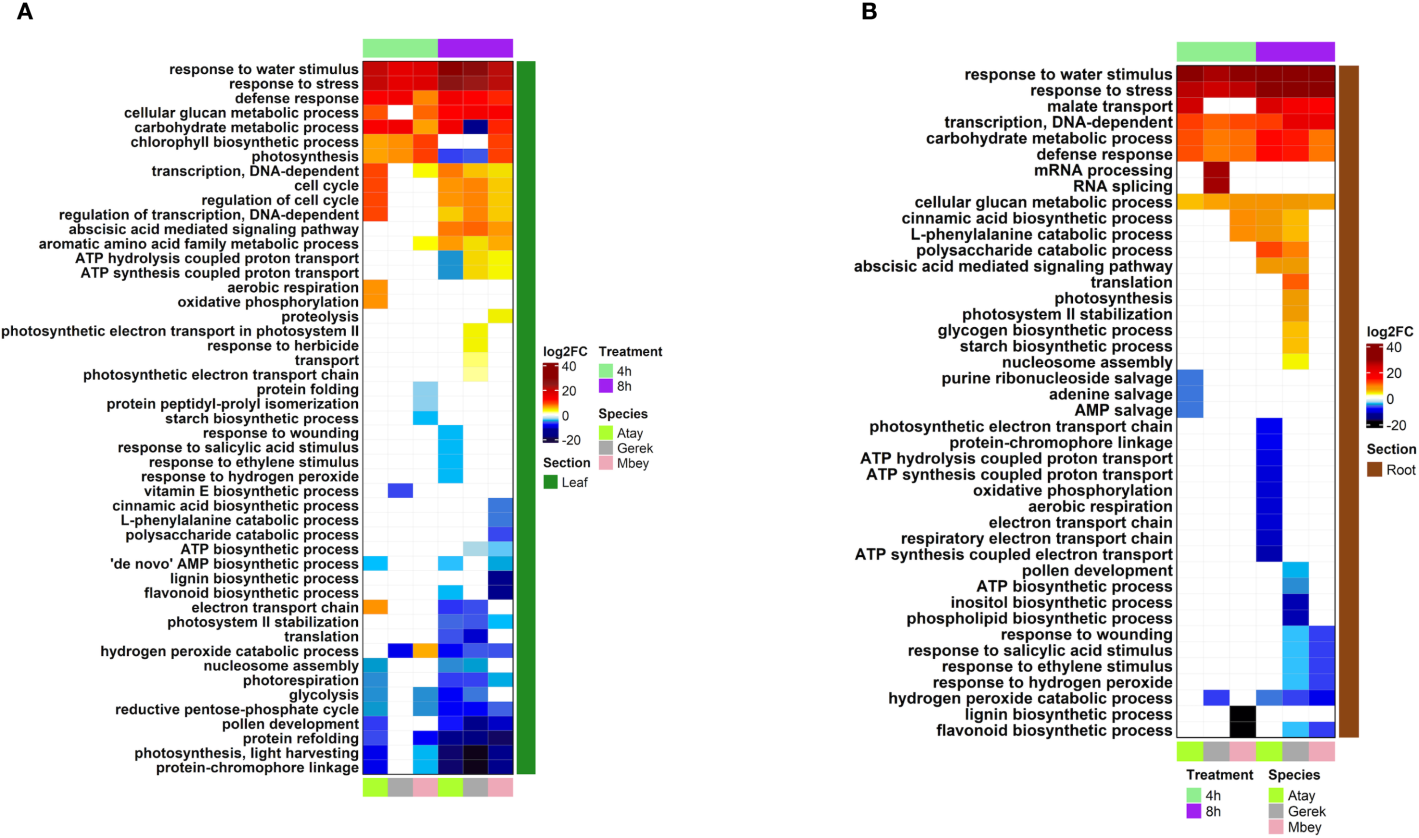
DEG analysis of biological functions in leaf **(A)** and root **(B)** tissues of drought-tolerant (Gerek 79, Müfitbey) and drought-sensitive (Atay 85) cultivars under 4- and 8-hour drought stress compared to control conditions. Red and blue indicate higher and lower expression values, respectively. Cultivars are color-coded as Atay 85 (green), Gerek 79 (grey), and Müfitbey (pink). Significant DEGs were identified at FDR ≤ 0.05.

**Figure 7:**
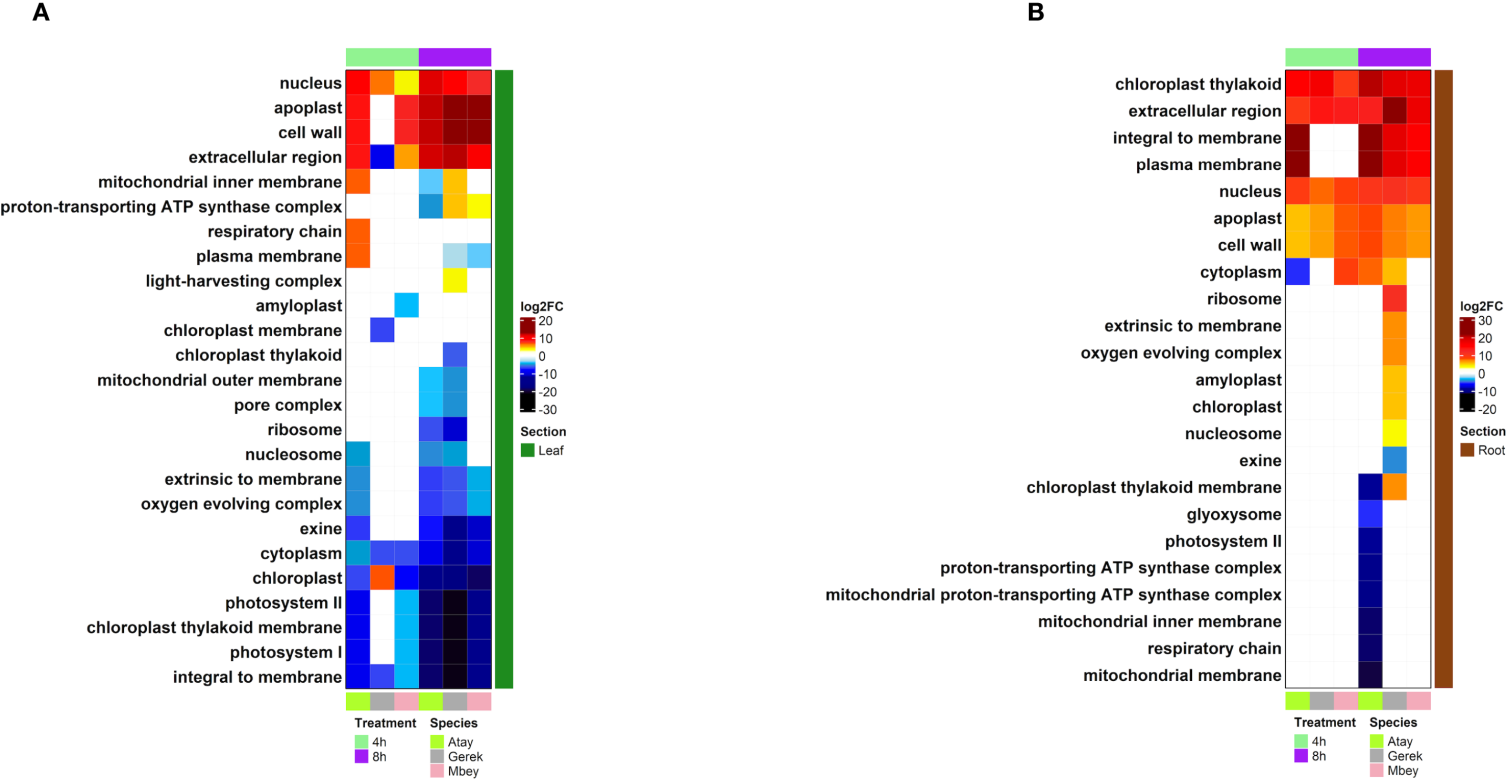
DEG analysis of cellular components in leaf **(A)** and root **(B)** tissues of drought-tolerant (Gerek 79, Müfitbey) and drought-sensitive (Atay 85) cultivars under 4- and 8-hour drought stress compared to control conditions. Red and blue indicate higher and lower expression values, respectively. Cultivars are color-coded as Atay 85 (green), Gerek 79 (grey), and Müfitbey (pink). Significant DEGs were identified at FDR ≤ 0.05.

**Figure 8:**
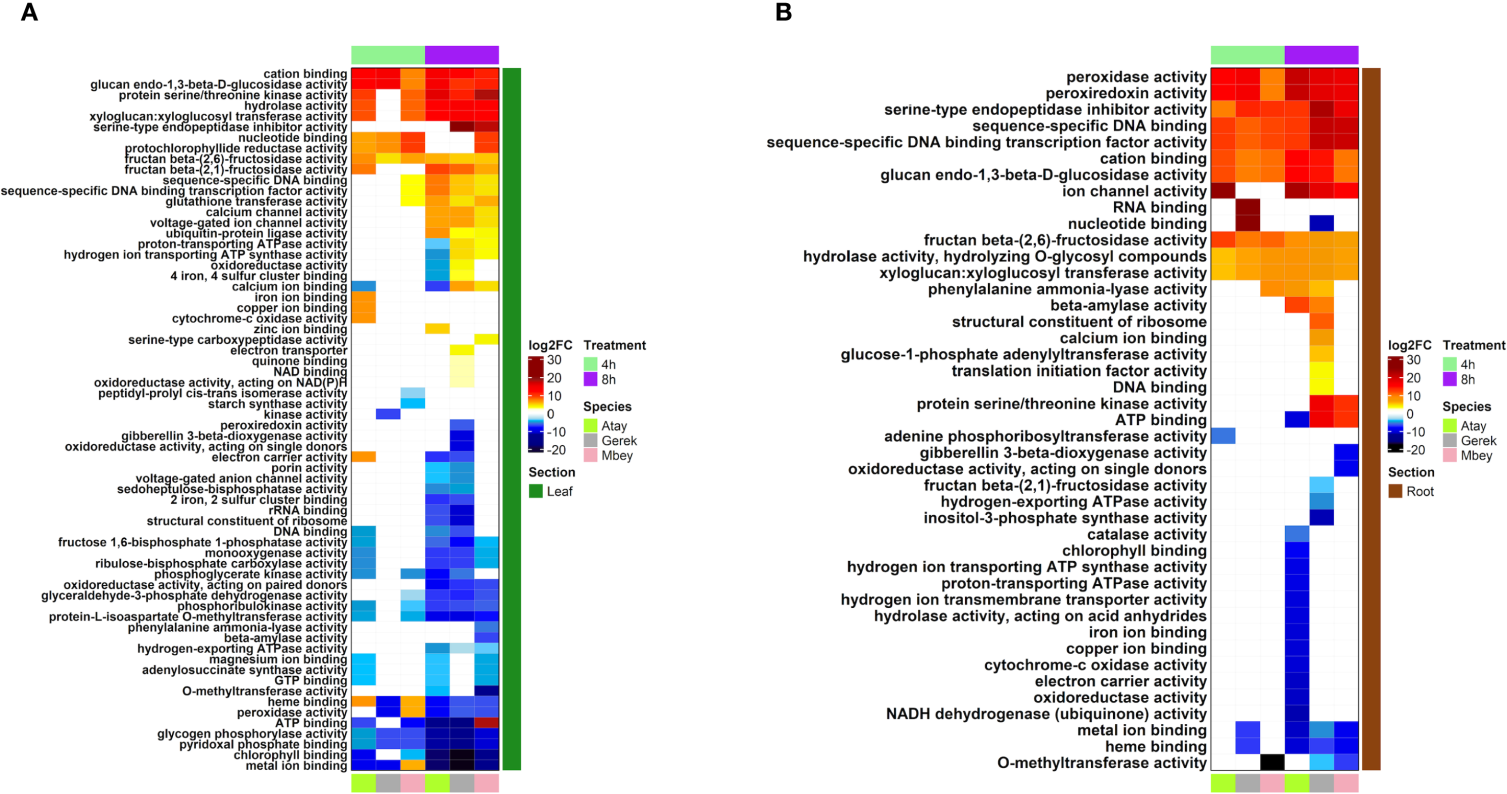
DEG analysis of molecular functions in leaf **(A)** and root **(B)** tissues of drought-tolerant (Gerek 79, Müfitbey) and drought-sensitive (Atay 85) cultivars under 4- and 8-hour drought stress compared to control conditions. Red and blue indicate higher and lower expression values, respectively. Cultivars are color-coded as Atay 85 (green), Gerek 79 (grey), and Müfitbey (pink). Significant DEGs were identified at FDR ≤ 0.05.

RNA-seq was performed on root and leaf tissues of the selected cultivars, Atay 85, Gerek 79, and Müfitbey, subjected to 4- or 8-hour shock dehydration or maintained under control conditions to assess transcript-level differences (**Figure 4A, B**). To further explore cultivar- and tissue-specific responses, Gene Ontology (GO) enrichment analysis was conducted on differentially expressed genes (DEGs). Significant DEGs were categorized into biological processes, molecular functions, and cellular components (**Figures 5**–**8**; **Supplementary Figures S3**-**S8**), enabling comparisons of conserved and unique drought-response mechanisms among cultivars.

The distribution of gene expression levels was more variable in leaf tissues than in roots. In Atay 85 roots under 8-hour drought stress (p < 0.01), upregulated genes were enriched in categories related to ion transport, including GO:0015078~hydrogen ion transmembrane transporter activity, GO:0015077~monovalent inorganic cation transmembrane transporter activity, and GO:0022890~inorganic cation transmembrane transporter activity. In contrast, the tolerant cultivar Müfitbey showed enrichment in biosynthetic and energy-related processes, such as GO:0034404~nucleobase, nucleoside, and nucleotide biosynthetic process, GO:0034654~nucleic acid biosynthetic process, GO:0016469~proton-transporting two-sector ATPase complex, GO:0045259~proton-transporting ATP synthase complex, and GO:0044271~nitrogen compound biosynthetic process. In Gerek 79, leaves under 8-hour drought stress, upregulated genes were associated with GO:0005506~iron ion binding, GO:0046906~tetrapyrrole binding, and GO:0009767~photosynthetic electron transport chain (**Figures 4**, **6**-**8**).

At 4 hours of drought stress (**Figure 5A**), 37 genes were commonly regulated across all three cultivars. Müfitbey had the highest number of unique genes (8), followed by Atay 85 (7) and Gerek 79 (3). Pairwise overlaps included 5 shared genes between Atay 85 and Gerek 79, 3 between Atay 85 and Müfitbey, and 3 between Gerek 79 and Müfitbey. At 8 hours of drought stress (**Figure 5B**), the number of shared genes across all cultivars decreased to 15, indicating more divergent responses over time. Atay 85 had 9 unique genes, Gerek 79 had 10, and Müfitbey had 2. Pairwise overlaps included 3 genes between Atay 85 and Gerek 79, 4 between Gerek 79 and Müfitbey, while Atay 85 and Müfitbey shared none. Unique DEGs for sensitive and tolerant cultivars are listed in **Supplementary Tables S1** and **S2**.

#### 3.3.1 Distinct transcriptomic strategies drive drought adaptation in wheat cultivars

We then investigated the cultivar-specific responses to drought stress in leaf and root tissues. Atay 85 (drought-sensitive) exhibited severe metabolic suppression, marked by strong activation of stress-response pathways, ABA signalling, and carbohydrate metabolism, alongside significant downregulation of photosynthesis and energy production—indicating an inability to maintain energy balance. Gerek 79 (moderately tolerant) showed a more balanced response, with moderate defense activation and limited suppression of ATP biosynthesis, sustaining partial metabolic activity under stress. In contrast, Müfitbey (highly tolerant) maintained energy production, enhanced carbohydrate metabolism, and activated structural reinforcement and antioxidant pathways, ensuring superior drought resilience (**Figure 6A**).

Root transcriptomes also reflected these differences (**Figure 6B**). Stress-responsive pathways, including “response to stress” and “ABA-mediated signalling,” were strongly upregulated across cultivars, while energy production processes such as oxidative phosphorylation and electron transport were downregulated. Atay 85 showed the sharpest decline in ATP biosynthesis, whereas Gerek 79 maintained moderate energy output and reinforced root structure via lignin and flavonoid biosynthesis. Müfitbey demonstrated the most effective strategy, preserving energy homeostasis and activating antioxidant defenses, enabling better root survival under drought. These findings highlight distinct molecular mechanisms underlying drought tolerance, with Müfitbey integrating metabolic stability and stress defense most effectively.

#### 3.3.2 Drought stress drives divergent cellular and metabolic reprogramming in wheat cultivars

Analysis of Differentially Expressed Genes showed that drought stress triggers distinct transcriptional and metabolic adjustments in wheat cultivars with varying tolerance levels. As shown in **Figure 7A**, genes associated with extracellular regions (cell wall, apoplast) and the nucleus are strongly upregulated, reflecting structural reinforcement and stress signalling. Conversely, photosynthesis-related components, including Photosystem I, Photosystem II, and chloroplast-associated proteins, are markedly downregulated—a common drought adaptation strategy to minimize energy-demanding processes.

Cultivar-specific responses reveal contrasting survival mechanisms. Atay 85 (drought-sensitive) exhibits severe metabolic suppression, compromising energy production and increasing vulnerability. Gerek 79 (moderately tolerant) maintains a balance between stress responses and energy metabolism, enabling moderate resilience. In contrast, Müfitbey (highly tolerant) sustains robust energy production and stress defense, conferring superior drought tolerance in both leaves and roots (**Figure 7A, B**).

Further analysis (**Figure 7B**) highlights differential regulation of cellular structures, including the plasma membrane, extracellular matrix, nucleus, and chloroplast. Mitochondrial components such as the respiratory chain and ATP synthase complex display mixed regulation, indicating cultivar-specific metabolic adjustments. Interestingly, Atay 85 reinforces root structure while maintaining basal metabolism, Gerek 79 prioritizes membrane transport and moderate energy conservation, whereas Müfitbey downregulates mitochondrial and photosynthetic activity, possibly invoking dormancy-like mechanisms for survival. These results highlight how genetic and metabolic diversity drives differential drought adaptation strategies among wheat cultivars.

#### 3.3.3 Wheat cultivars employ distinct functional strategies for drought survival

Wheat cultivars adopt contrasting functional strategies to withstand drought, ranging from osmoprotection to metabolic dormancy. These strategies reflect cultivar-specific genetic programs that balance energy conservation, water retention, oxidative stress mitigation, and long-term survival, with implications for drought resilience and recovery (**Figure 8A, B**).

Tolerant cultivars exhibit coordinated leaf-level adjustments that complement root signalling to preserve photosynthetic integrity (**Figure 8A**). Genes encoding PSII core proteins (psbA, psbD), light-harvesting complexes (LHCBs), and RuBisCO activase are maintained or upregulated, safeguarding photochemistry and carbon assimilation. ABA signalling in guard cells (PYR/PYL–SnRK2) ensures timely stomatal closure, reducing water loss while preventing photoinhibition via regulated non-photochemical quenching. Aquaporins sustain mesophyll hydration despite reduced stomatal conductance. Antioxidant systems, supported by the ascorbate–glutathione cycle, maintain low ROS levels, while osmoprotectants stabilize proteins and membranes. Structural reinforcements through cell wall remodeling, lignin deposition, and cuticular wax biosynthesis delay senescence and maintain tissue integrity. These adjustments preserve photosynthetic efficiency (ΦPSII, Fv′/Fm′), water-use efficiency, and chlorophyll content, enabling rapid post-drought recovery. In contrast, the sensitive cultivar Atay 85 shows sharp downregulation of photosynthetic genes, leading to early PSII damage and reduced CO_2_ fixation. ABA-mediated stomatal control is inefficient, causing excessive transpiration followed by severe dehydration. Weak antioxidant defenses and osmolyte production result in ROS accumulation, lipid peroxidation, and chlorophyll degradation. Structural maintenance pathways are underrepresented, accelerating leaf curling, rolling, and senescence. Consequently, photosynthetic and hydraulic decline is rapid, and recovery is poor (**Figure 8A**).

Root transcriptomes reveal cultivar-specific metabolic reprogramming under drought (**Figure 8B**). Tolerant genotypes activate gene modules sustaining water and ion homeostasis. Aquaporins (PIP, TIP) and cell wall-modifying enzymes (expansins, XTHs) enhance root hydraulic conductivity, while ABA biosynthesis genes (NCEDs) and signalling components (PYR/PYL–SnRK2) ensure precise shoot–root communication. Ethylene production is moderated, possibly via ACC deaminase pathways, favoring continued root growth. Ion transporters (SOS1, NHX1, HKT-like) and K^+^ retention systems maintain ionic balance, while osmoprotectant biosynthesis (proline, trehalose, raffinose family oligosaccharides) preserves turgor. Strong antioxidant capacity (SOD, APX, CAT, GR, GSTs) mitigates ROS accumulation, protecting meristematic integrity. Conversely, Atay 85 exhibits weak induction of aquaporins and wall-modifying genes, reducing root hydraulic conductivity. Erratic ABA–ethylene regulation impairs root growth and signalling to leaves. Ion homeostasis collapses due to poor Na^+^ exclusion and K^+^ retention, while limited osmolyte and antioxidant production predispose roots to oxidative injury. These deficiencies trigger early hydraulic failure, amplifying stress signals to the shoot.

Cultivar-specific drought survival strategies: **Figure 8A, B** illustrate contrasting drought tolerance mechanisms among wheat cultivars:

Atay 85 (sensitive) maintains moderate photosynthesis and reinforces structural components (cell wall, apoplast) while relying on rapid ROS detoxification and transcriptional regulation. However, weak root hydraulics, limited osmolyte production, and poor root–shoot coordination lead to oxidative damage, impaired photosynthesis, and accelerated senescence under prolonged stress. Gerek 79 (moderately tolerant) adopts an energy-conservative strategy, suppressing photosynthesis while enhancing osmotic adjustment via sugar metabolism (notably fructans). Root responses include strong induction of aquaporins (PIP, TIP), cell wall remodeling enzymes (expansins, XTHs), ABA biosynthesis and signalling (NCEDs, PYR/PYL–SnRK2), ion transporters (SOS1, NHX1, HKT-like), osmoprotectant synthesis, and antioxidant systems. These traits sustain root hydraulics, ion homeostasis, and oxidative balance, ensuring effective root–shoot signalling. Müfitbey (highly tolerant) employs a dormancy-like strategy, shutting down photosynthesis and mitochondrial activity while activating hormonal signalling and protein stabilization pathways for long-term survival. Root responses mirror Gerek 79 but with stronger hormonal regulation, maximizing drought endurance at the cost of slower post-stress recovery.

These strategies involve trade-offs, as Müfitbey’s dormancy ensures survival during prolonged drought but delays recovery, potentially affecting harvest timing, Gerek 79’s osmoprotective balance supports moderate yields under intermittent drought and Atay 85’s limited tolerance risks severe yield loss under water deficits, emphasizing the need for targeted cultivar selection in water-limited environments. Tolerance in Gerek 79 and Müfitbey is driven by early activation of root hydraulics, ion transport, and hormonal signalling, synchronized with leaf-level protective programs whereas in Atay 85, poor root–shoot coordination amplifies stress effects, accelerating functional decline. This tissue-resolved framework identifies key genetic and physiological nodes for breeding drought-resilient wheat.

### 3.4 KEGG pathway enrichment analysis

**Figure 9:**
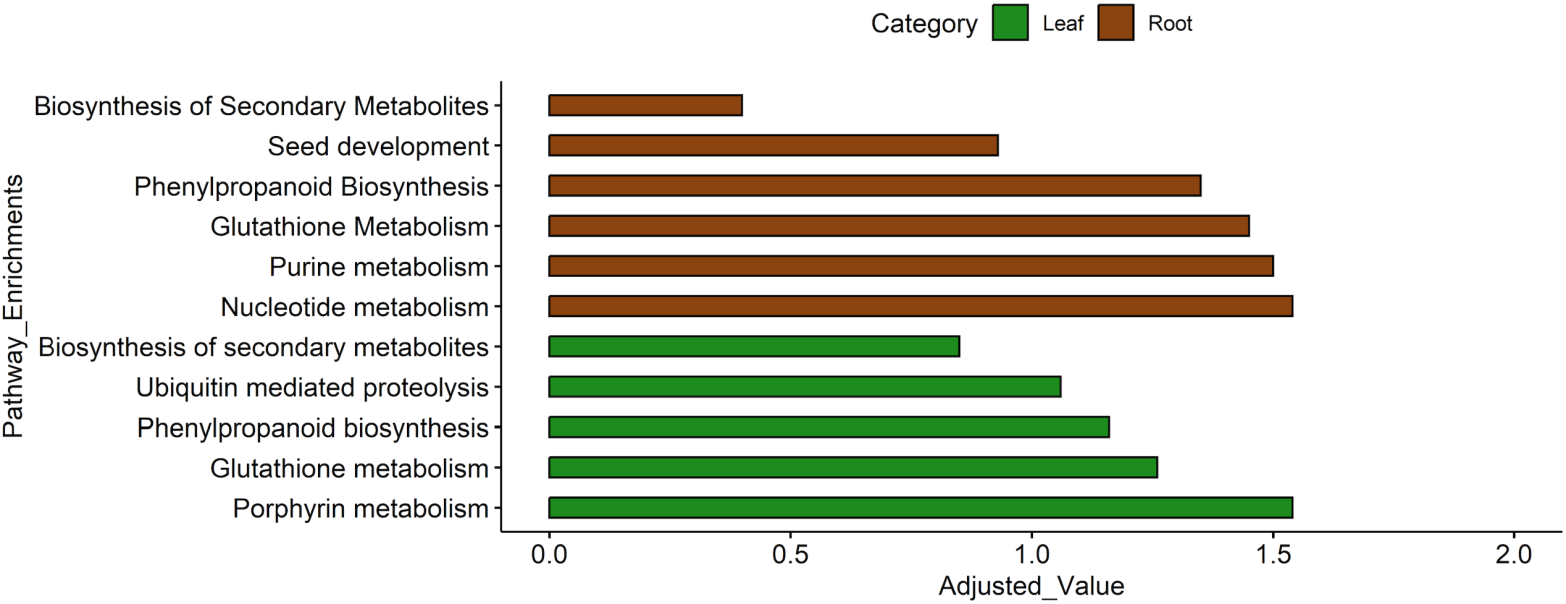
KEGG pathway enrichment analysis, based on significant DEGs in root and leaf tissues. This bar graph represents pathway enrichments of both upregulated and downregulated genes in leaf (green) and root (brown) tissues, based on adjusted values. The x-axis shows the adjusted values, indicating the significance of enrichment for each pathway. The y-axis lists different biological pathways enriched in the two tissues.

Pathway enrichment analysis of significantly differentially expressed genes revealed tissue-specific drought responses (**Figure 9**).

In roots, enrichment of secondary metabolite biosynthesis and phenylpropanoid pathways suggests enhanced production of protective compounds (e.g., flavonoids, lignin) that reinforce root structure and improve drought tolerance. Glutathione metabolism plays a central role in detoxifying drought-induced reactive oxygen species (ROS), while nucleotide and purine metabolism supports DNA/RNA synthesis and energy balance, ensuring root growth and repair under stress. Interestingly, pathways linked to seed development were also upregulated, possibly reflecting a survival mechanism to secure future reproduction. In leaves, enrichment of porphyrin metabolism indicates an effort to sustain chlorophyll synthesis and maintain photosynthetic activity despite water deficit. Activation of ubiquitin-mediated proteolysis facilitates protein turnover by removing damaged proteins and recycling amino acids—an essential adaptive mechanism during stress. Similar to roots, glutathione and phenylpropanoid pathways in leaves contribute to ROS scavenging and structural reinforcement, protecting cellular integrity. Together, roots and leaves exhibit activation of secondary metabolite biosynthesis, highlighting a broad stress-adaptive strategy. However, roots prioritize metabolic adjustments for growth maintenance, oxidative stress reduction, and developmental adaptation, whereas leaves focus on preserving photosynthesis, regulating protein turnover, and maintaining cellular homeostasis under drought.

### 3.5 qRT-PCR validation of differentially expressed genes under drought stress

To validate the RNA-seq results, twelve drought-responsive genes identified from the DEG analysis were selected for expression profiling using qRT-PCR. Although fold-change values differed between RNA-seq and qRT-PCR, the overall expression trends were consistent, confirming the reliability of the RNA-seq data.

**Figure 10:**
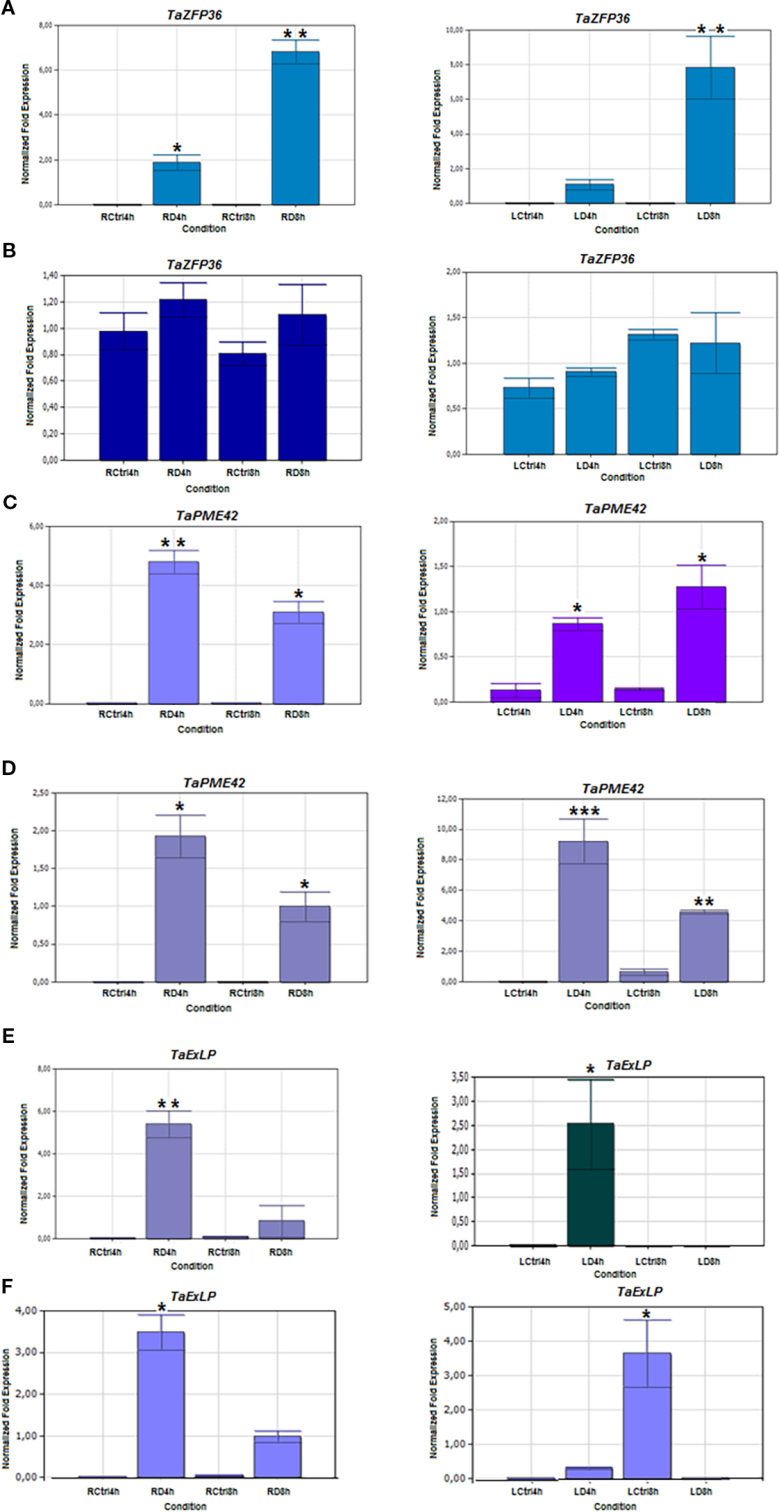
Expression patterns of Zinc finger CCCH domain-containing protein 36 (TaZFP36), pectinesterase/pectinesterase inhibitor 42 (TaPME42), and Extensin-like protein (TaExLP) genes in 4- and 8-hour drought-stressed root and leaf tissues. **(A, C, E)** Drought- tolerant Müfitbey); **(B, D, F)** Drought-sensitive Atay 85 cultivar. LCtrl, Leaf Control; LD, Leaf Drought; RCtrl, Root Control; RD, Root Drought. Error bars correspond to the standard error of the means. Statistical significance was determined using a t-test, with p ≤ 0.05 represented by a single asterisk (*) and p ≤ 0.01 by double asterisks (**), indicating significant differences in expression between the control and drought groups.

**Figure 11:**
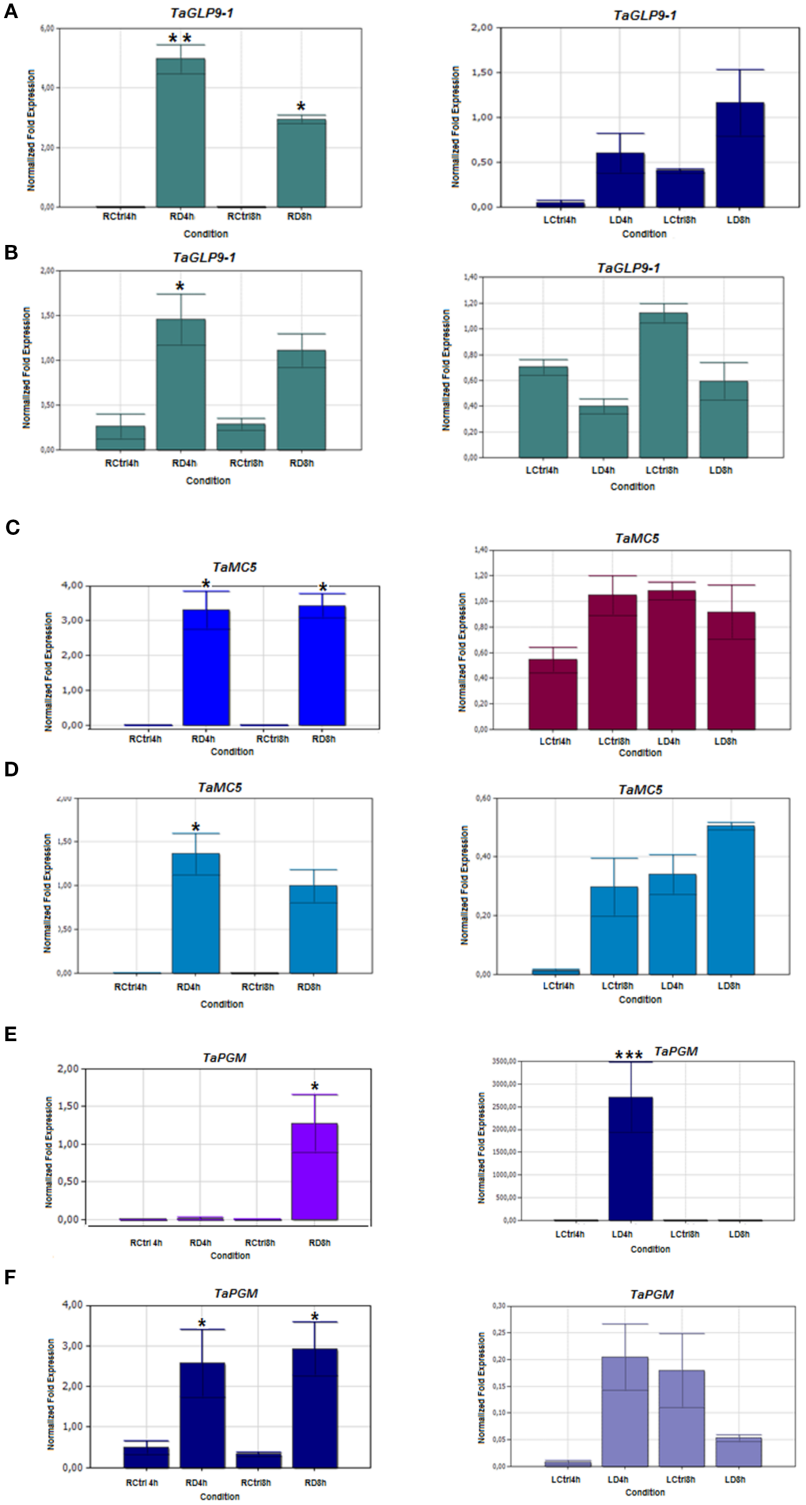
Expression patterns of Germin-like protein 9-1 (TaGLP 9-1), Metacaspase-5 (TaMC5), and Phosphoglycerate/bisphosphoglycerate mutase (TaPGM) genes in 4- and 8-hour drought-stressed root and leaf tissues. **(A, C, E)** Drought- tolerant Müfitbey; **(B, D, F)** Drought-sensitive Atay 85 cultivar. LCtrl, Leaf Control; LD, Leaf Drought; RCtrl, Root Control; RD, Root Drought. Error bars correspond to the standard error of the means. Statistical significance was determined using a t-test, with p ≤ 0.05 represented by a single asterisk (*) and p ≤ 0.01 by double asterisks (**), indicating significant differences in expression between the control and drought groups.

**Figure 12:**
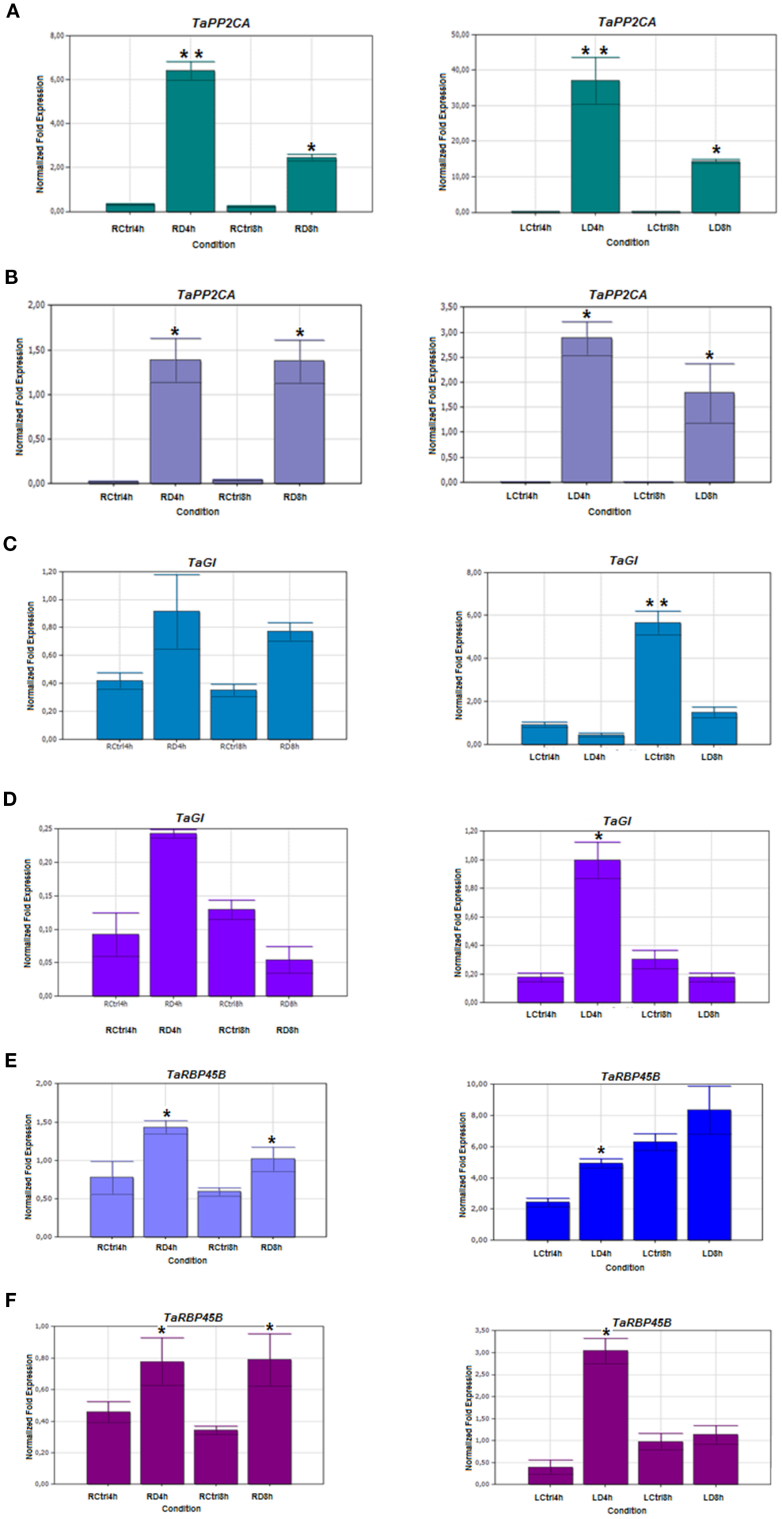
Expression pattern of Serine/threonine protein phosphatase 2A (TaPP2CA), GIGANTEA (TaGI), and Polyadenylate-binding protein (TaRBP45B) in root and leaf tissues under 4- and 8-hour drought-stress. **(A, C, E)** Drought- tolerant Müfitbey; **(B, D, F)** Drought-sensitive Atay 85 cultivar. LCtrl, Leaf Control; LD, Leaf Drought; RCtrl, Root Control; RD, Root Drought. Error bars correspond to the standard error of the means. Statistical significance was determined using a t-test, with p ≤ 0.05 represented by a single asterisk (*) and p ≤ 0.01 by double asterisks (**), indicating significant differences in expression between the control and drought groups.

The selected genes represent key stress-related pathways, including cell wall remodeling, oxidative stress response, hormone signalling, and metabolic regulation. These include:

TaPME42 (pectinesterase/pectinesterase inhibitor 42), TaExLP (extensin-like protein), TaGLP9-1 (germin-like protein 9-1), TaZFP36 (CCCH-type zinc finger protein 36), TaMC5 (metacaspase-5), TaPGM (phosphoglycerate/bisphosphoglycerate mutase), TaPP2CA (protein phosphatase 2C), TaGI (GIGANTEA), TaRBP45B (RNA-binding protein), TaFER (ferritin), TaADT (arogenate dehydratase 5), and TaFBW2 (F-box protein). Expression was assessed in root and leaf tissues of drought-stressed and control plants. qRT-PCR validation confirmed differential expression of genes under drought stress (**Figures 10**–**12**).

TaZFP36 (zinc finger protein) was strongly induced in roots and leaves of the tolerant cultivar Müfitbey, but not in Atay 85. TaFER (ferritin) increased in leaves of both cultivars, peaking at 8 hours (**Supplementary Figure S9**). TaPME42 (pectinesterase) was consistently upregulated in roots and leaves of both cultivars. TaExLP (extensin-like protein) showed strong induction in roots of both cultivars but cultivar-specific patterns in leaves, higher in Atay 85 at 4 hours, reduced in Müfitbey.

TaGLP9-1 (germin-like protein) was upregulated in roots of both cultivars, but only Müfitbey showed increased expression in leaves. In defense responses, TaMC5 (metacaspase-5) was induced in roots of Müfitbey but unchanged in Atay 85. TaADT-5 (arogenate dehydratase) increased in leaves of both cultivars at 4 hours, with an eight-fold rise in Müfitbey at 8 hours, while Atay 85 declined (**Supplementary Figure S10**). TaPGM (Phosphoglycerate mutase) increased in roots of Atay 85 at both time points and in leaves of Müfitbey at 4 hours. TaPP2CA was significantly upregulated in roots and leaves of both cultivars, peaking at 4 hours in most tissues. Finally, TaGI (GIGANTEA) decreased in Müfitbey leaves at 8 hours but increased in Atay 85 at 4 hours. TaRBP45B (RNA-binding protein) was induced in roots of both cultivars and transiently in leaves at 4 hours. TaFBW2 (F-box protein) showed cultivar-specific induction, suggesting a role in ubiquitin-mediated proteolysis and stress signalling.

## 4 Discussion

Drought stress severely impacts plant growth and can significantly reduce wheat yields, especially in cultivated areas. To better understand the mechanisms of drought response in hexaploid wheat, it is essential to study gene expression patterns in both tolerant and sensitive genotypes. Although several studies have explored comparative transcriptome responses to drought in various crop species, the specific molecular mechanisms in *Triticum aestivum* cultivars with differing tolerance levels—tolerant, mildly tolerant, and sensitive—remain underexplored.

In this study, we investigated drought-responsive gene expression in three T. aestivum cultivars: drought-tolerant (Müfitbey), mildly tolerant (Gerek 79), and drought-sensitive (Atay 85). Since gene expression changes are more pronounced under acute water deficiency than under progressive drought, we applied a shock drought stress model, as previously described (Ergen et al., 2009).

**Genotype-specific antioxidant patterns reveal divergent ROS management strategies under drought stress.**

Drought stress triggered pronounced, genotype- and tissue-specific changes in antioxidant enzyme activities, highlighting distinct adaptive mechanisms in Atay 85, Gerek 79, and Müfitbey. Superoxide dismutase (SOD), which catalyzes the dismutation of superoxide radicals (O_2_•^-^) into hydrogen peroxide (H_2_O_2_), showed differential activity patterns. In tolerant genotypes, SOD activity increased under stress, facilitating continuous conversion of superoxide radicals and placing greater demand on downstream scavengers such as peroxidase (POX), ascorbate peroxidase (APX), and catalase (CAT) to maintain ROS homeostasis (Devi et al., 2012; Sallam et al., 2019).

Integrated analysis of antioxidant enzyme activities (SOD, POX, APX, CAT) alongside TBARS accumulation revealed genotype-dependent drought responses. Müfitbey maintained a coordinated antioxidant profile, with moderate increases in SOD, POX, and CAT in leaves and stable APX activity, resulting in the lowest TBARS levels. This suggests efficient ROS detoxification and supports its classification as the most drought-tolerant genotype (Haouari et al., 2013). Gerek 79 exhibited organ-specific responses: while root CAT and APX activities were suppressed, leaf SOD, POX, and CAT were strongly induced. However, this activation did not prevent a significant rise in TBARS, particularly at 8 h, indicating oxidative damage. These findings position Gerek 79 as moderately tolerant, capable of partial defense but susceptible to lipid peroxidation under prolonged stress. Atay 85 showed strong induction of CAT and APX in roots and elevated APX in leaves, indicating an active enzymatic response. Nevertheless, high TBARS accumulation in leaves suggests that its antioxidant defenses were insufficient to fully mitigate oxidative injury (Chakraborty and Pradhan, 2012). Thus, Atay 85 can be considered moderately sensitive, with robust but suboptimal protective responses.

Overall, these results highlight genotype-specific strategies: Müfitbey shows superior tolerance, Gerek 79 intermediate resilience, and Atay 85 reduced efficiency in balancing antioxidant activity and oxidative damage.

**Role of transcription factors and gene expression patterns in drought tolerance.**

Our findings revealed distinct physiological and molecular responses in both root and leaf tissues, with observable variations between the 4- and 8-hour time points and among the three cultivars. These responses also differed notably from those observed in their respective control groups.

In leaf tissues, a consistent trend of decreased gene expression was observed for cellular processes such as protein refolding and metabolic pathways like photorespiration as drought stress duration increased (8 hours) across all three cultivars. Comparative transcriptome profiling provided valuable insights into the complexity of drought stress responses at the molecular level. Analysis of RNA-seq data indicated that metabolic processes related to gene expression were predominantly activated in response to both 4- and 8-hour drought stress.

Furthermore, the drought-tolerant cultivars (Müfitbey and Gerek 79) exhibited increased expression levels of genes associated with protein binding, metabolic processes, and cellular functions, suggesting a greater adaptive capacity to drought stress compared to the sensitive cultivar Atay 85. Similar findings have been reported in Cucumis sativus L. under drought stress, where significant increases in gene expression were observed, particularly in metabolic processes, membrane-related functions, and catalytic activity (Wang C. T. et al., 2018).

Transcription factors (TFs) are key regulators of stress responses and are frequently targeted as candidate genes for improving stress tolerance (Moumeni et al., 2011). By binding directly to the promoters of target genes in a sequence-specific manner, TFs modulate the activation or repression of downstream genes in response to environmental stimuli (Ciarmiello et al., 2014). Therefore, identifying and characterizing stress-responsive TFs is essential for advancing molecular breeding strategies aimed at enhancing drought tolerance.

In our study, the sensitive cultivar exhibited over 25 differentially expressed TFs in leaf tissues under both 4- and 8-hour drought stress, but only four TFs were identified in root tissues. In contrast, the tolerant cultivar showed more than 80 TF transcripts in both leaves and roots after 4 hours of drought stress, with this number decreasing to 18 after 8 hours. These findings underscore the role of TFs in drought tolerance and suggest that multiple TF families contribute to the underlying resistance mechanisms. This is consistent with studies in other crops such as Hordeum vulgare, where TFs like HvWRKY12 and HvDRF1 have been implicated in mediating drought stress responses (Gürel et al., 2016).

**Metabolic and Energy Pathway Responses to Drought Stress in Tolerant and Sensitive Wheat Cultivars:**

Under 8-hour drought stress, leaf tissues exhibited decreased expression of genes involved in hydrogen peroxide catabolism, photorespiration, glycolysis, and photosystem II stabilization. This suggests a decline in photosynthetic efficiency and overall metabolic activity, likely due to stress-induced cellular damage. In contrast, the upregulation of genes related to carbohydrate metabolism, defence responses, and glucan metabolism in both leaf and root tissues under 4- and 8-hour drought stress indicates an adaptive strategy aimed at maintaining cellular energy balance and enhancing stress resilience.

In the drought-sensitive cultivar Atay 85, root tissues under 8-hour drought stress showed reduced expression of genes associated with oxidative phosphorylation, aerobic respiration, ATP hydrolysis and synthesis, and the electron transport chain (ETC). This points to a disruption in energy production, potentially impairing root functionality and increasing susceptibility to drought stress (**Figure 5A**).

Cultivar-specific expression patterns in leaf tissues further illustrate these differences. Atay 85 undergoes a near-complete shutdown of energy production under drought conditions, rendering it highly vulnerable. Gerek 79 (moderately tolerant) maintains partial metabolic activity while managing stress, whereas Müfitbey (highly tolerant) sustains a balanced response between energy production and stress adaptation, making it the most drought-resilient cultivar.

A similar trend was observed in root tissues. Müfitbey showed upregulation of genes involved in defence responses and secondary metabolite biosynthesis, including lignin, flavonoid, and phospholipid pathways. This indicates activation of structural reinforcement and antioxidant mechanisms, contributing to improved root survival and overall plant resilience (**Figure 5A, B**).

These findings underscore Müfitbey’s superior drought tolerance, achieved through efficient energy management and activation of protective pathways in both leaf and root tissues.

### 4.1 Metal ion binding plays a role in drought response

Our study revealed drought-induced upregulation of metal ion-binding genes in both leaf and root tissues of T. aestivum, including those involved in heme, 2Fe-2S cluster, and zinc, iron, and copper binding. Proteins such as AtTZF1–3 are known to regulate plant growth and stress responses (Wang et al., 2008).

In Arabidopsis, 11 CCCH-type TZFs with plant-specific motifs were identified (Wang et al., 2008; Pomeranz et al., 2010). AtTZF1–6 and AtTZF9 are involved in ABA signaling, seed germination, and PAMP-triggered immunity, and localize to stress granules and processing bodies, influencing post-transcriptional and epigenetic regulation (Anderson and Kedersha, 2009). Gain-of-function AtTZF1 lines show enhanced drought and cold tolerance via ABA/GA modulation (Lin et al., 2011).

In wheat, 269 TaZFPs exhibit stress-responsive cis-elements and tissue-specific expression, suggesting roles in growth and abiotic stress adaptation (Wu et al., 2022). Notably, TaZFP36 was significantly upregulated in both tissues of drought-tolerant cultivars, but not in the sensitive cultivar Atay 85, indicating its potential role in drought tolerance, consistent with AtTZF1 studies.

We also observed regulation of TaFER, a ferritin gene involved in iron storage and oxidative stress response (Wu et al., 2022). Ferritin accumulation in chloroplasts is triggered by various stressors including ozone, ethylene, and iron overload (Fobis-Loisy et al., 1995; Van Wuytswinkel and Briat, 1995; Murgia et al., 2002). Our qRT-PCR results showed differential TaFER expression in leaf tissues of tolerant and sensitive cultivars, highlighting its role in iron regulation and drought adaptation.

### 4.2 Cell wall proteins clearly play a role in drought response

Differential gene expression analysis under drought stress revealed several cell wall-related genes, including Beta-galactosidase 1, Glucose-6-phosphate/phosphate-translocator, TaExLP4, TaExLP6, TaGLP9-1, and lignin biosynthesis genes. Three were selected for further analysis due to elevated expression: TaPME49, TaExLP, and TaGLP9-1 (**Supplementary Table S3**).

TaPME49, involved in pectin demethylesterification, affecting cell wall plasticity (Kohli et al., 2015) showed increased in drought-stressed leaf tissues of both tolerant and sensitive cultivars, but decreased in roots, indicating tissue-specific regulation (Al-Qsous et al., 2004; Micheli, 2001). TaExLP, a hydroxyproline-rich glycoprotein (HRGP) involved in development and stress responses (Kurepa and Smalle, 2023) was strongly induced in roots at 4 hours, with higher early expression in sensitive cultivars. Its suppression in tolerant ones suggests a role in early stress signaling rather than long-term adaptation. TaGLP9-1, a Germin-like proteins contribute to ROS-mediated defence and are associated with abiotic stress responses (Christensen et al., 2004; Cevher-Keskin et al., 2019), was upregulated in drought-tolerant leaf tissues, supporting its role in resistance, consistent with proteomic data (Faghani et al., 2015).

### 4.3 Defence response proteins in drought stress

ABA regulates drought responses and interacts with salicylic acid (SA) pathways to modulate defence against pathogens (Gupta et al., 2020; Cao et al., 2011) with defence-related genes, including TaADT5 and TaMC5, were upregulated in drought-stressed leaf tissues. TaADT5, a key enzyme in lignin biosynthesis and possibly anthocyanin production (Corea et al., 2012a; Corea et al., 2012b; Muhammad et al., 2023) showed increased expression in tolerant cultivars suggests a role in drought resilience. Related genes ADT1 and ADT3 also contribute to anthocyanin synthesis under stress (Chen et al., 2016). TaMC5, a metacaspase involved in programmed cell death (PCD) and defence (Uren et al., 2000; Valandro et al., 2020) was elevated in 8-hour drought-stressed root and leaf tissues of Müfitbey, indicating its role in drought tolerance via PCD regulation.

### 4.4 Drought stress activates carbohydrate degradation-related genes

Phosphoglycerate/Bisphosphoglycerate Mutase (PGM) is a key enzyme in the glycolysis pathway, catalyzing the transfer of phosphate groups among the three carbon atoms of phosphoglycerate. PGM also dephosphorylates and activates Actin-Depolymerizing Factor 1 (ADF1), a protein that governs the re-modelling of the actin cytoskeleton which is essential for maintaining cell structure and intracellular transport under stress conditions (Oslund et al., 2017).

In our study, TaPGM expression significantly increased in the roots of the drought-sensitive cultivar Atay 85 after 8 hours of stress (**Figure 9E, F**). In contrast, the tolerant cultivar showed early induction in leaf tissue at 4 hours, with no notable change at 8 hours (**Figure 7E, F**). This suggests a dual role for TaPGM in drought response: late root-specific induction in sensitive plants may reflect a delayed compensatory mechanism, while early leaf expression in tolerant plants indicates a proactive adaptation strategy. Overall, TaPGM expression timing and tissue specificity appear to be key factors in drought tolerance in T. aestivum.

### 4.5 Involvement of ABA-related genes in drought stress

Protein Phosphatase 2A (PP2A), a serine/threonine phosphatase, plays diverse roles in biotic and abiotic stress responses (Pais et al., 2009). It negatively regulates ABA signaling and influences ABA-dependent gene expression and light-mediated nitrate reductase activation (Chen et al., 2015; Creighton et al., 2017).

In rice (Oryza sativa), all five catalytic subunit genes (OsPP2A-1–5) are upregulated under salinity stress (Yu et al., 2003). Similarly, salt stress elevates StPP2Ac1–3 transcripts in potato leaves, and okadaic acid inhibition confirms PP2A’s positive role in stress regulation (Pais et al., 2009). In wheat, TaPP2Ac-1 accumulates under water deficit, and its overexpression in tobacco enhances drought tolerance (Xu et al., 2007). In Arabidopsis, PP2A-C5 overexpression activates chloride channels (AtCLCa, AtCLCc), improving ion sequestration and tolerance to salt and drought (Hu et al., 2017; Balasubramanian et al., 2022).

In our study, TaPP2CA expression was significantly upregulated in both leaf and root tissues of tolerant and sensitive cultivars after 4 and 8 hours of drought stress (**Figure 12A, B**). This early and sustained induction suggests a key role in drought perception and ABA-mediated signalling.

### 4.6 Regulation of photoperiodism in drought stress

Prolonged drought (8 hours) led to downregulation of genes involved in photosynthesis, light harvesting, photosystem I stabilization, and photorespiration in both tolerant and sensitive cultivars, indicating reduced photosynthetic activity due to stress-induced damage and energy conservation.

One key regulator is GIGANTEA (GI), a multifunctional protein involved in circadian rhythm, photoperiodism, phytochrome B signaling, and flowering (Krahmer et al., 2018). GI is modulated by environmental cues such as cold, hydrogen peroxide, blue light, and karrikin (Waters et al., 2014; Krahmer et al., 2018), and stabilizes ADO3 and ADO1/ZTL, regulating CONSTANS (CO) in the long-day flowering pathway. GI also enhances salinity tolerance via SOS2 interaction and induces EARLY FLOWERING (ELF) under drought (Kim et al., 2013; Riboni et al., 2013). Mutations in GI improve oxidative and freezing stress tolerance through CDF upregulation (Fornara et al., 2015). GI also promotes the “Drought Escape” (DE) response, accelerating flowering under drought (Bader et al., 2023).

In our study, TaGI expression was reduced in 8-hour drought-stressed leaf tissues of the tolerant cultivar Müfitbey (**Figure 12C, D**), supporting its negative role in drought tolerance, consistent with GI knockout studies.

RNA-Binding Protein RBP45B, part of the hnRNP family, binds poly(A) tails and is involved in mRNA maturation and translation initiation. RBPs are widely upregulated under abiotic stress (salt, drought, heat, cold, ozone, hypoxia, flooding), highlighting their role in stress tolerance (Yan et al., 2022). TaRBP45B was induced in drought-stressed root tissues at both time points, with cultivar-specific expression in leaves (**Figure 12E, F**), suggesting a positive role in drought adaptation.

The three wheat cultivars examined in this study exhibit distinct physiological and molecular responses to drought stress. Atay 85, the drought-sensitive cultivar, shows pronounced metabolic suppression and a decline in ATP production. This is accompanied by poor coordination between root and leaf responses, resulting in oxidative damage and premature senescence. In contrast, Gerek 79, which displays moderate tolerance, manages to conserve energy while activating osmoprotective mechanisms and reinforcing structural components. Müfitbey, the highly tolerant cultivar, maintains metabolic stability and initiates robust hormonal and antioxidant responses. It appears to adopt a dormancy-like strategy, allowing it to endure prolonged drought conditions more effectively.

Across all cultivars, photosynthesis-related genes are generally downregulated under drought stress, while genes associated with stress responses and structural integrity are upregulated. Mitochondrial activity varies among cultivars, reflecting species-specific metabolic adjustments. These findings highlight the diverse drought adaptation strategies employed by wheat and point to potential genetic targets for breeding more resilient cultivars.

This study identifies candidate drought-responsive genes in wheat (e.g., TaZFP36, TaFER, TaPP2CA, TaGI, TaRBP45B) and highlights cultivar-specific strategies of tolerance. These genes provide targets for functional validation and genome editing to improve drought resilience. They also represent potential molecular markers that could be incorporated into breeding programs for selecting drought-tolerant genotypes. Integrating transcriptomic insights with breeding tools could support sustainable wheat production under water-limited conditions.

## Data Availability

The original contributions presented in the study are publicly available. This data can be found at the National Center for Biotechnology Information (NCBI) using accession number PRJNA1015138.
