# Supplementary figures and images for: Deciphering drought-response in wheat (Triticum aestivum): physiological, biochemical, and transcriptomic insights into tolerant and sensitive cultivars under dehydration shock
Source: Front Plant Sci. 2025 Oct 27;16:1649378. doi: 10.3389/fpls.2025.1649378 (PMC12598786; doi:10.3389/fpls.2025.1649378)

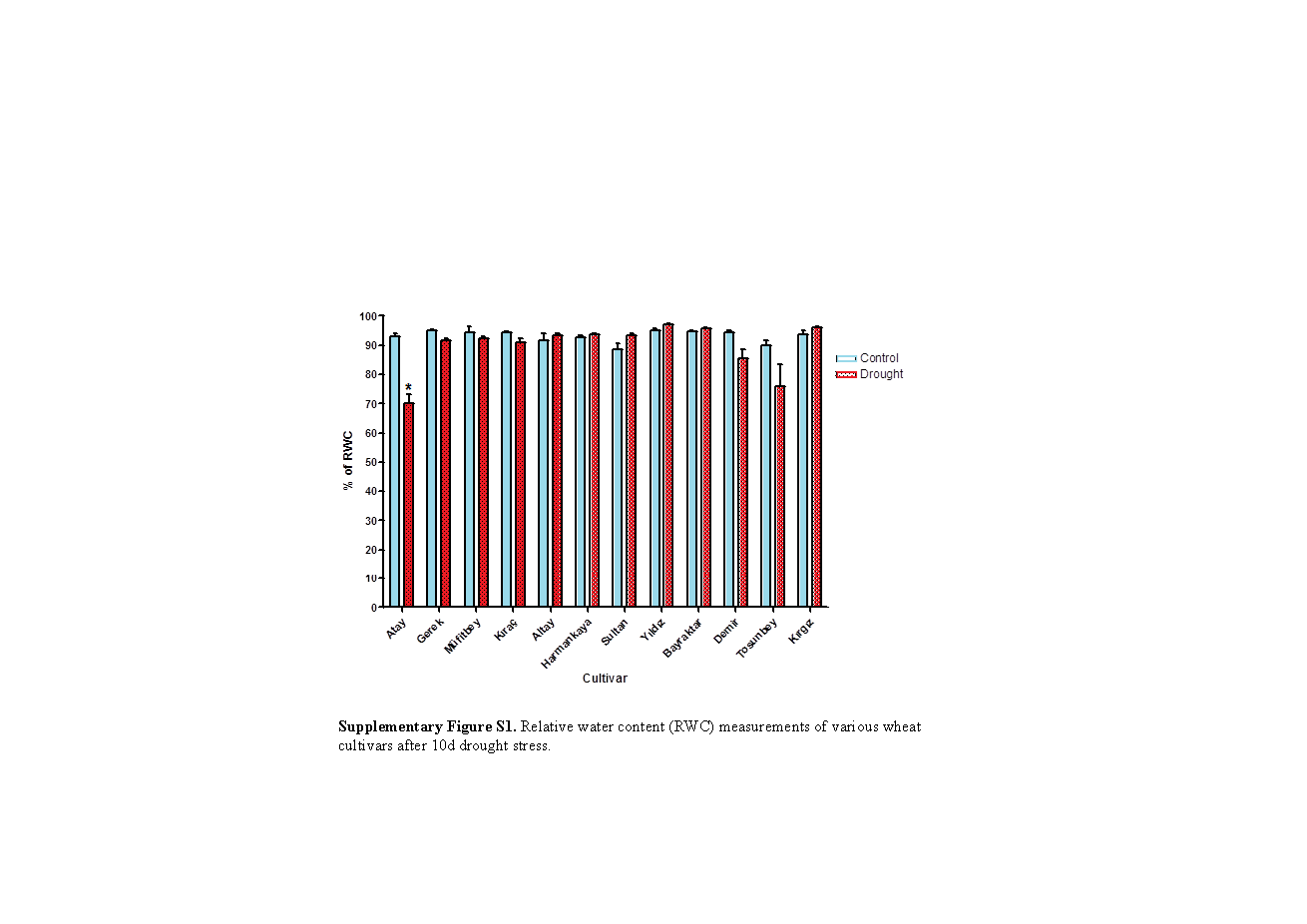

Supplement: Supplementary file 1 [file Image1.tiff]

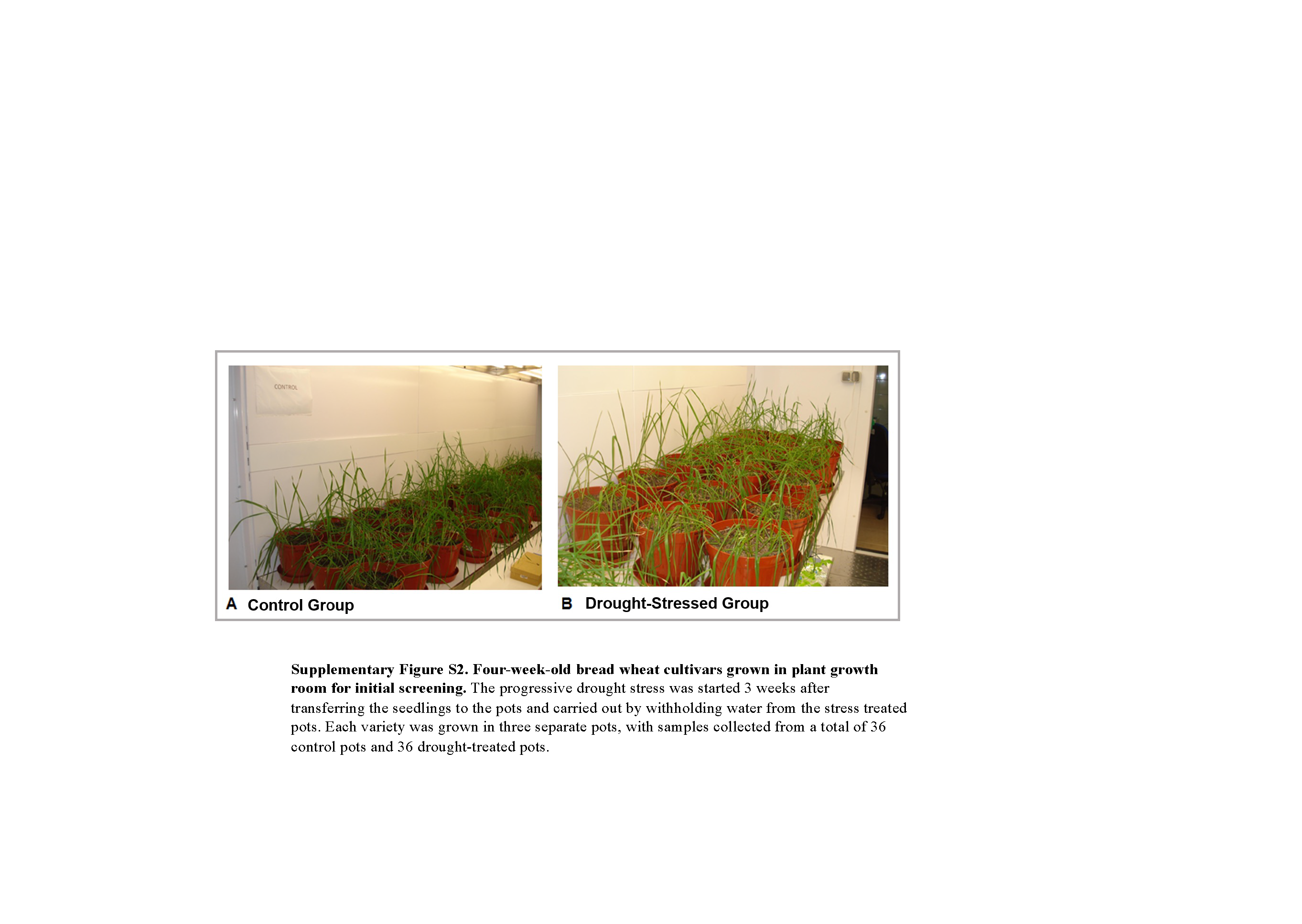

Supplement: Supplementary file 2 [file Image2.tiff]

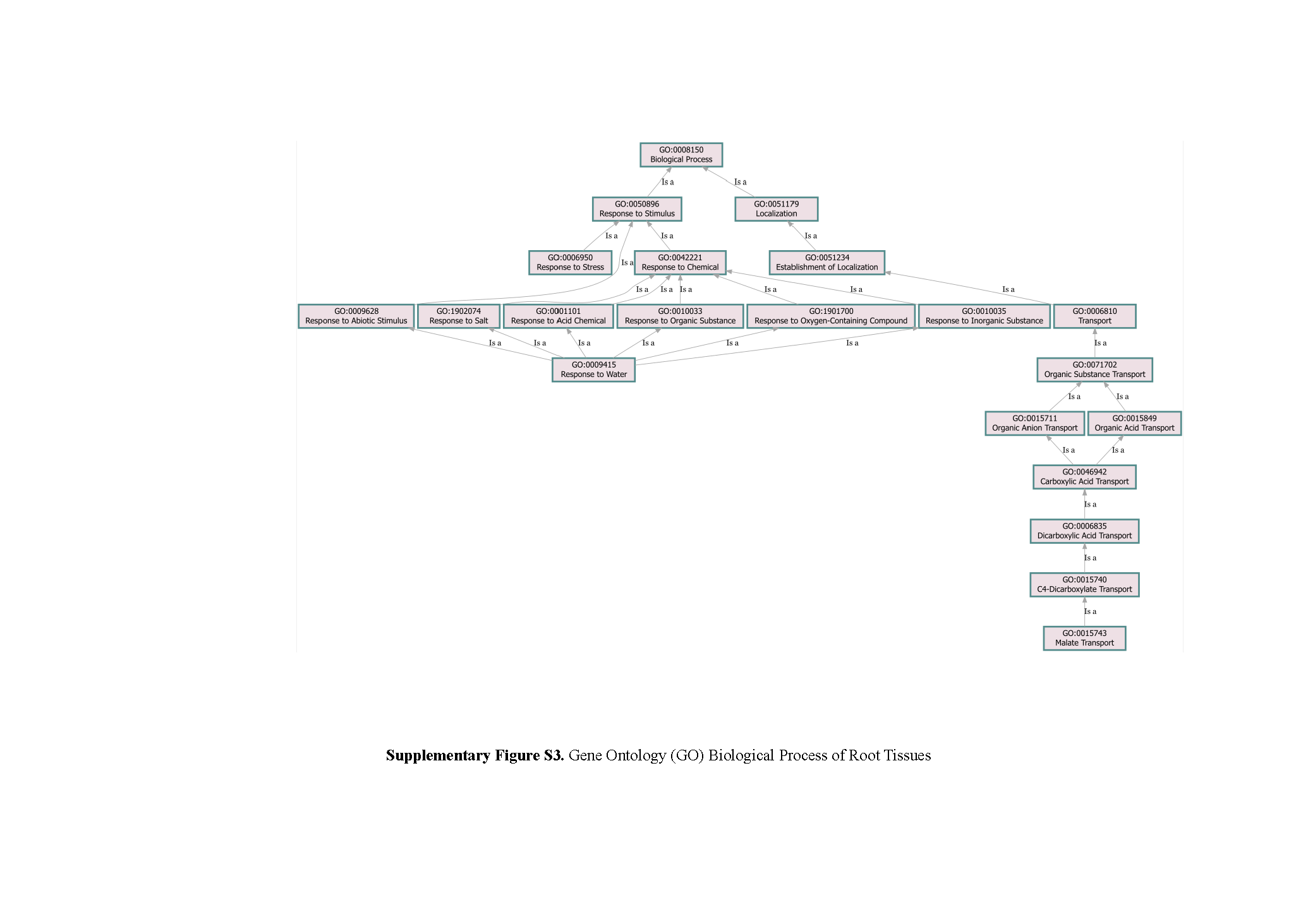

Supplement: Supplementary file 3 [file Image3.tiff]

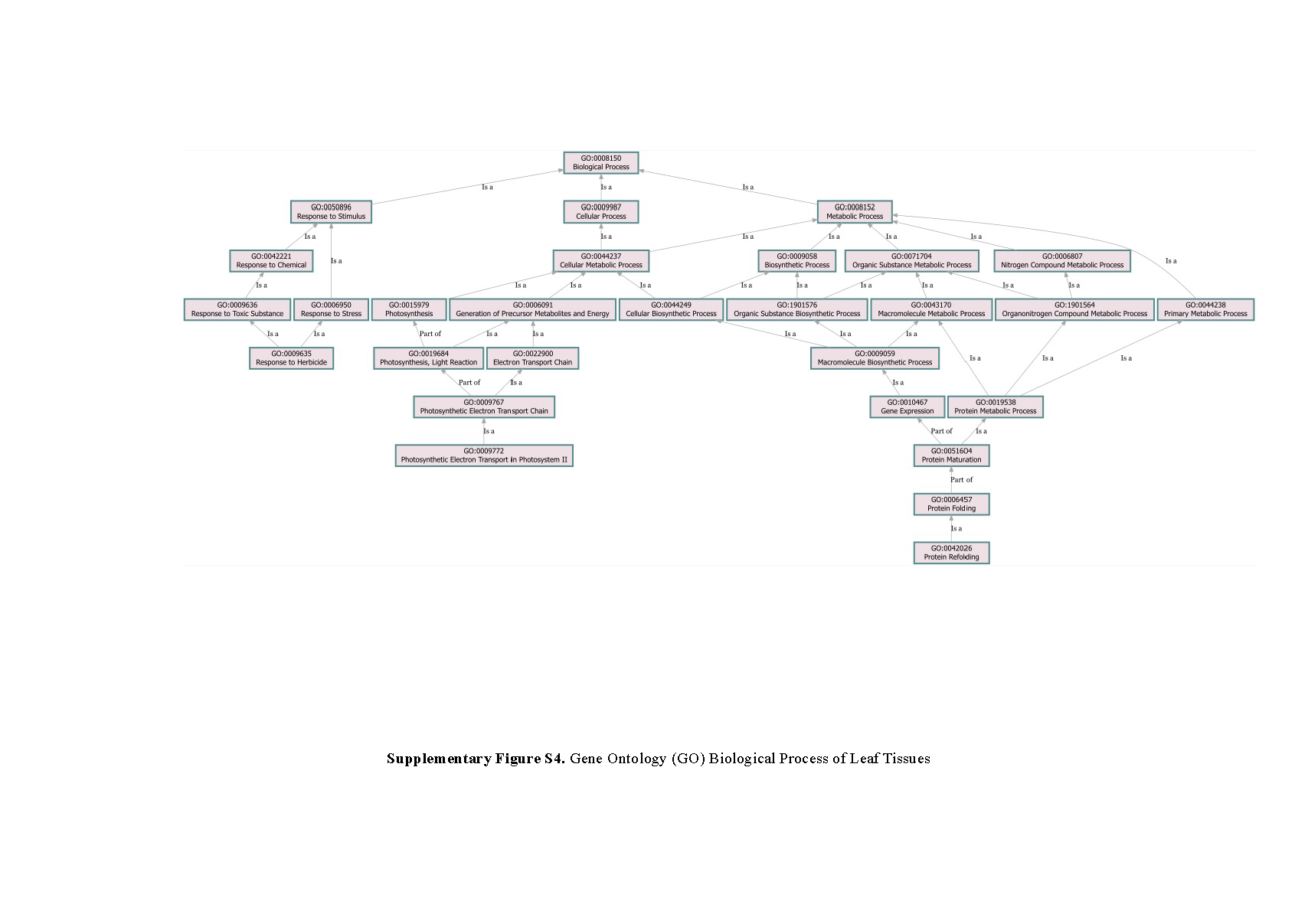

Supplement: Supplementary file 4 [file Image4.tiff]

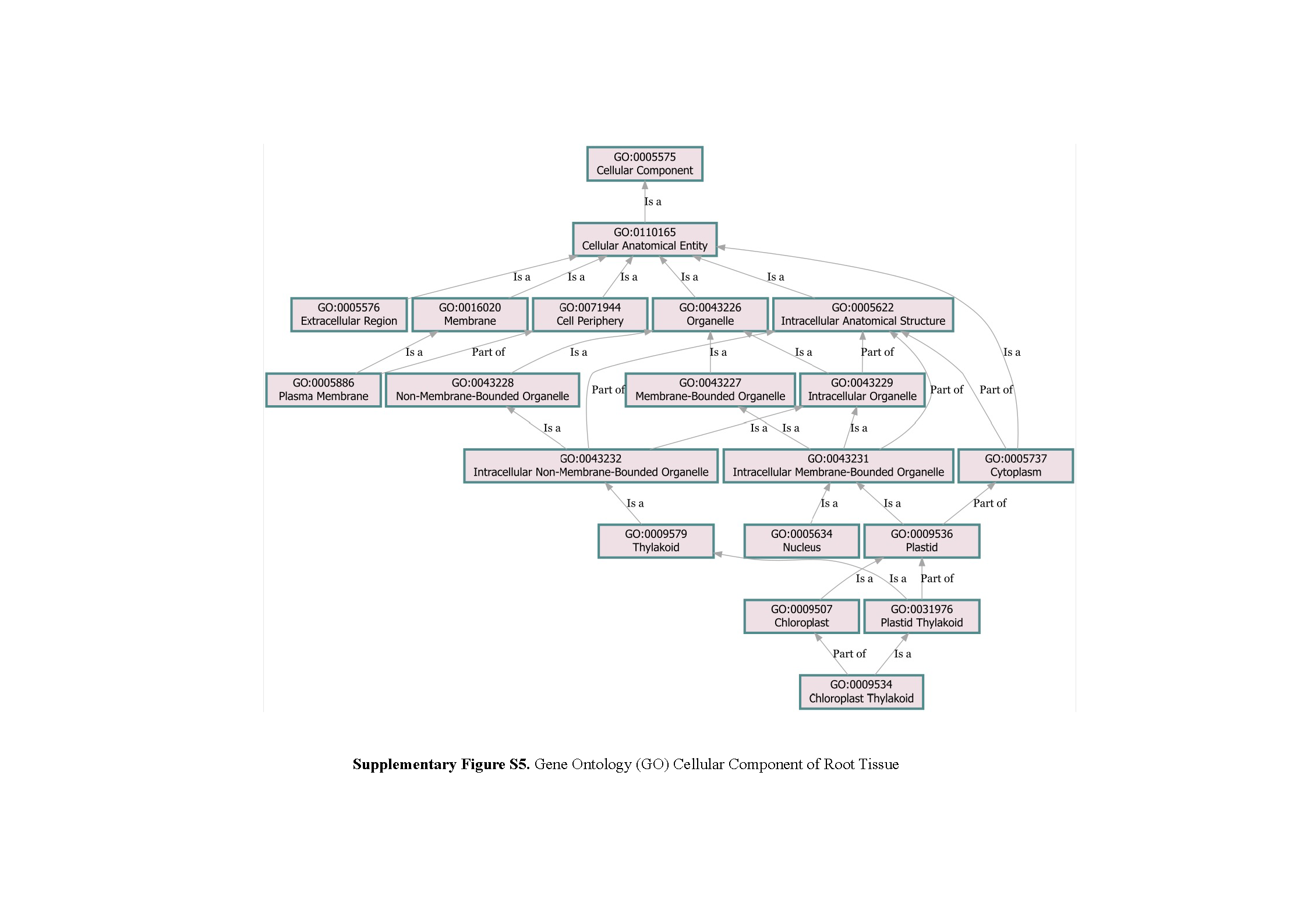

Supplement: Supplementary file 5 [file Image5.tiff]

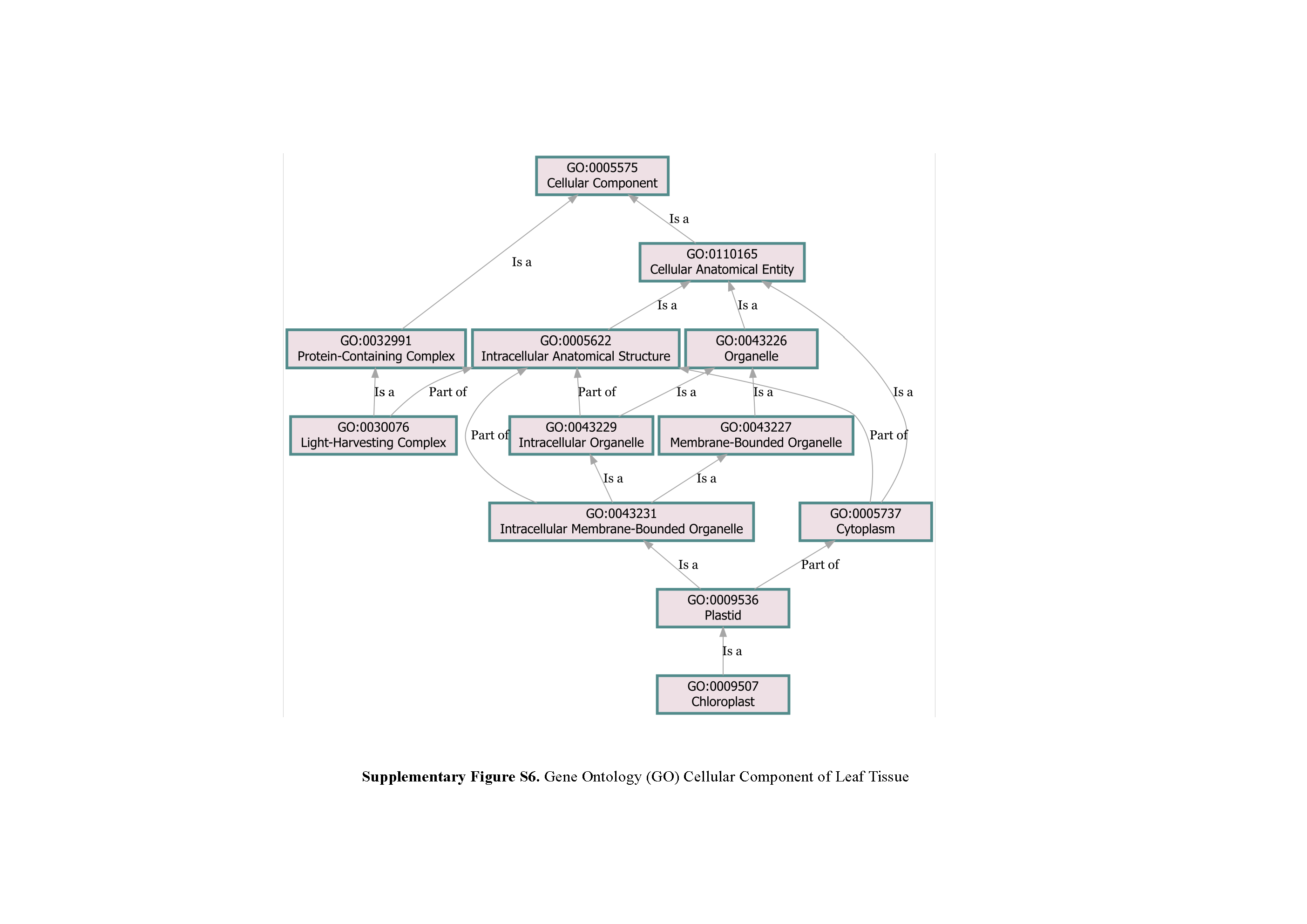

Supplement: Supplementary file 6 [file Image6.tiff]

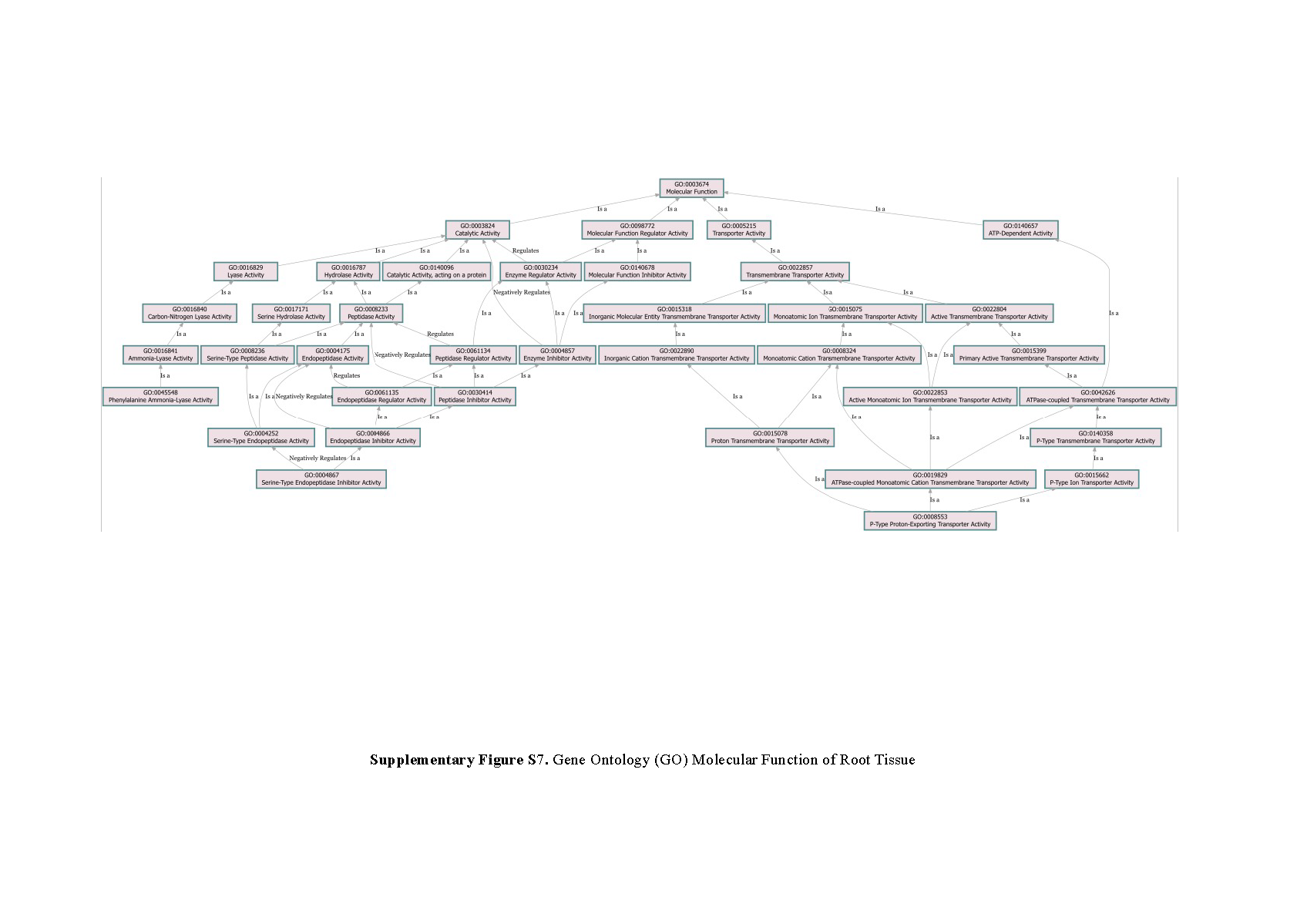

Supplement: Supplementary file 7 [file Image7.tiff]

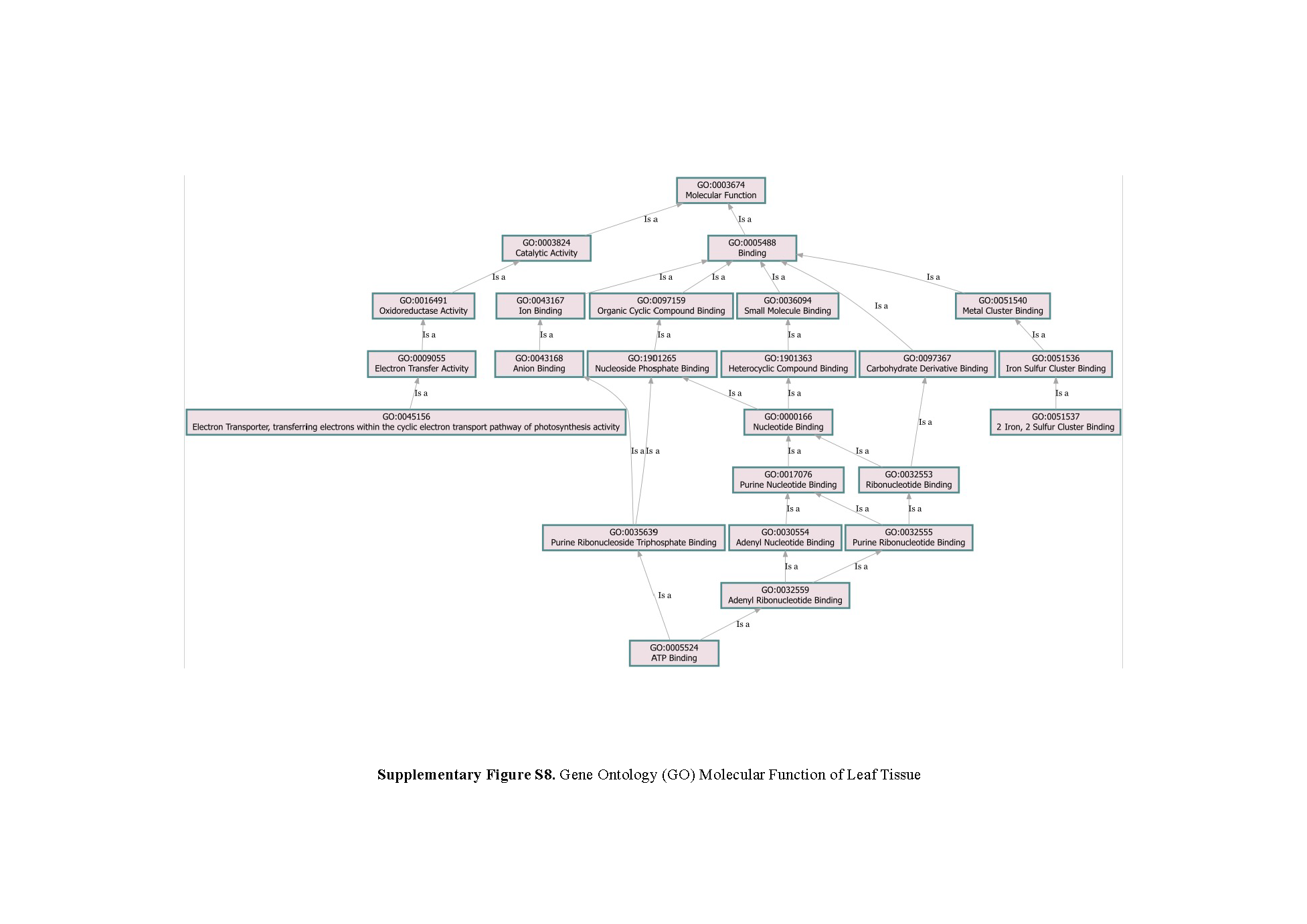

Supplement: Supplementary file 8 [file Image8.tiff]

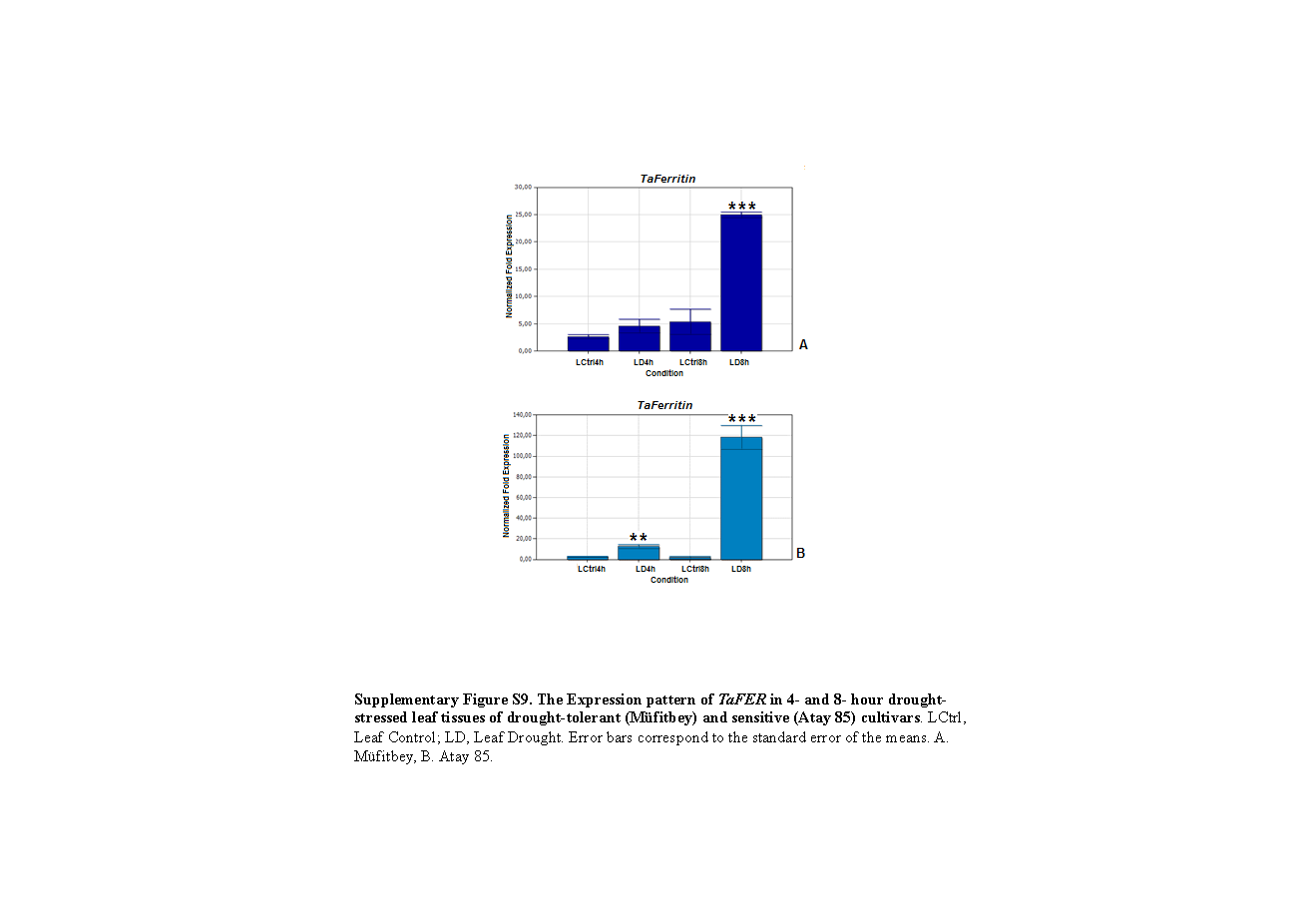

Supplement: Supplementary file 9 [file Image9.tiff]

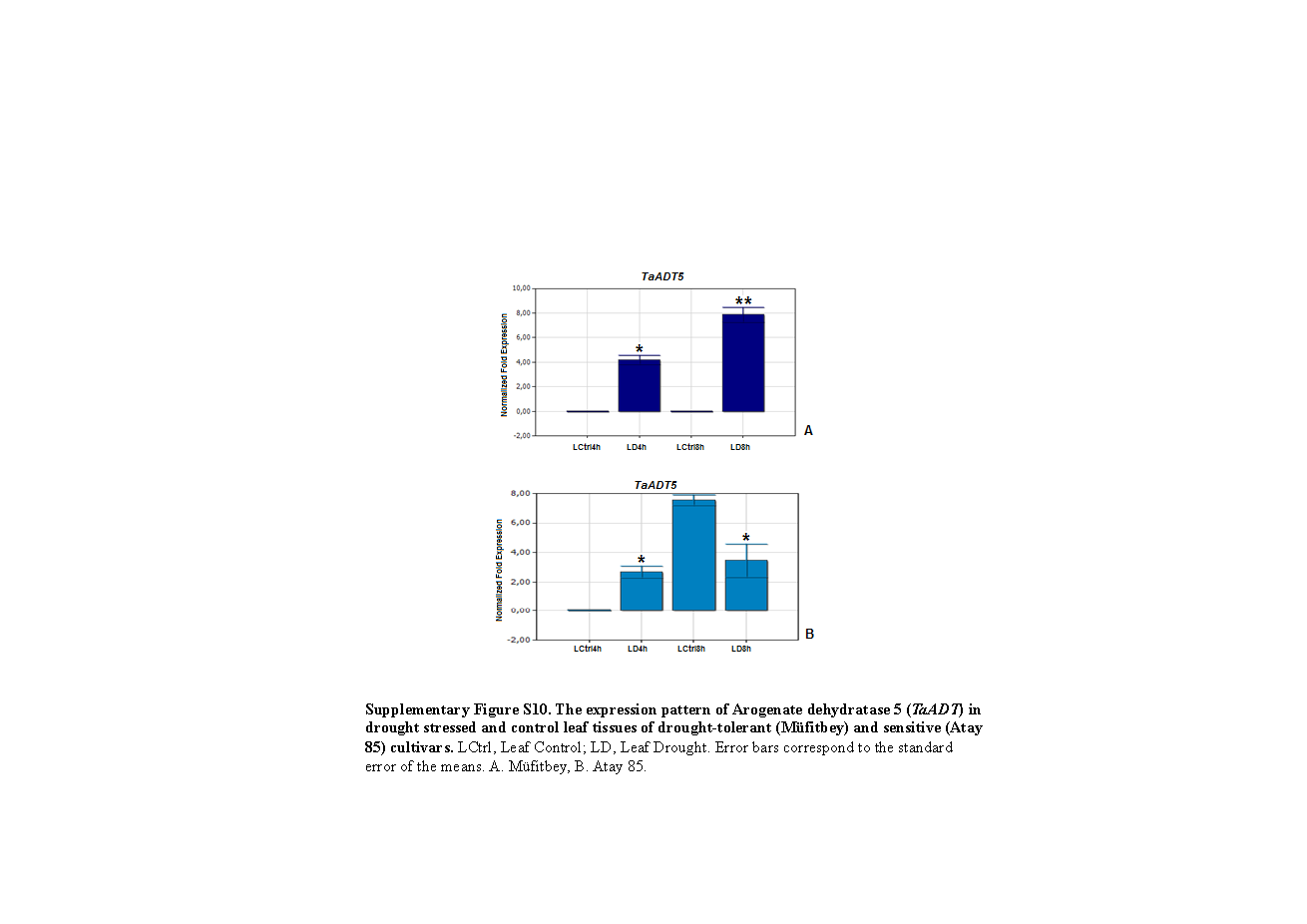

Supplement: Supplementary file 10 [file Image10.tiff]
