# Supplementary material for: Deciphering drought-response in wheat (Triticum aestivum): physiological, biochemical, and transcriptomic insights into tolerant and sensitive cultivars under dehydration shock
Source: Front Plant Sci. 2025 Oct 27;16:1649378. doi: 10.3389/fpls.2025.1649378 (PMC12598786; doi:10.3389/fpls.2025.1649378)
Supplement: Supplementary file 11 [file Table1.docx]

**Supplementary Table S1.** Unique Differentially Expressed Genes (DEGs) in leaf tissues of susceptible Atay 85 and tolerant Gerek 79 and Müfitbey cultivars.

| **Susceptible Leaf** | **Tolerant Leaf** |
| --- | --- |
| COX1 | PRX1 |
| atpB | GA3ox2-2 |
| ZFP1* | ndhH |
| OMT1 | HBP1C* |
| H2A3* | IBB2* |
| MT-CYB | ha1 |
| PDI | HBP1B* |
|  | atpA |
|  | CBP3 |
|  | HBP1A* |
|  | EM |
|  | WSSI-2 |
|  | FKBP70 |
|  | OMT2 |

**COX1** – Cytochrome c oxidase subunit I; **atpB** – ATP synthase subunit beta; **ZFP1** – Zinc Finger Protein 1; **OMT1**–O-methyltransferase; **H2A3**–Histone H2A type 3; **MT-CYB** – Mitochondrially encoded cytochrome b; **PDI** – Protein Disulfide Isomerase; **PRX1** – Peroxiredoxin 1; **GA3ox2-2** – Gibberellin 3-beta-dioxygenase 2-2; **ndhH** – NAD(P)H dehydrogenase subunit H; **HBP1C** – HMG-box transcription factor 1C; **IBB2** – Importin beta subunit 2; **ha1** – Proton-ATPase subunit A1; **HBP1B**– HMG-box transcription factor 1B; **atpA** – ATP synthase subunit alpha; **CBP3** – Chloroplast biogenesis protein 3; **HBP1A** – HMG-box transcription factor 1A; **EM** – Early Methionine-labeled protein; **WSSI-2** – Wheat Stress-Responsive Gene 2; **FKBP70** – FK506-binding protein 70; **OMT2** – O-methyltransferase 2.

Genes marked with an asterisk (*) are wheat DEGs indicated by their name in the UniProt database.
