# Supplementary material for: Deciphering drought-response in wheat (Triticum aestivum): physiological, biochemical, and transcriptomic insights into tolerant and sensitive cultivars under dehydration shock
Source: Front Plant Sci. 2025 Oct 27;16:1649378. doi: 10.3389/fpls.2025.1649378 (PMC12598786; doi:10.3389/fpls.2025.1649378)
Supplement: Supplementary file 12 [file Table2.docx]

**Supplementary Table S2.** Unique Differentially Expressed Genes (DEGs) in roots of susceptible Atay 85 and tolerant Gerek 79 and Müfitbey.

| **DEGs in Susceptible Root** | **DEGs in Tolerant Root** |
| --- | --- |
| APT1 | U2AF65A |
| MT-CYB | ha1 |
| ND4 | OMT2 |
| COX1 | GA3ox2-2 |
| MI25* | PER1* |
| YMF19 | HBP1C* |
| ATPA | OMT1 |
| CAT1 | AAIP* |
| psbB | RPL35 |
|  | PSBO |
|  | RAFTIN1A |
|  | 1-FEHw2 |
|  | AGP-L |
|  | H32* |
|  | IF1A* |
|  | MIPS |

**APT1** – Adenine Phosphoribosyltransferase 1; **MT-CYB** – Cytochrome b; **ND4** – NADH Dehydrogenase Subunit 4; **COX1** – Cytochrome c Oxidase Subunit I; **MI25** – ATP Synthase Protein MI25; **YMF19** – Putative ATP Synthase Protein YMF19; **ATPA** – ATP Synthase Subunit Alpha; **CAT1** – Catalase 1; **psbB** – Photosystem II CP47 Reaction Center Protein; **U2AF65A** – Splicing Factor U2AF Large Subunit A; **ha1** – Hemagglutinin Subunit 1; **OMT2** – Caffeic Acid 3-O-Methyltransferase 2; **GA3ox2-2** – Gibberellin 3-Beta-Dioxygenase 2-2; **PER1** – Period Circadian Regulator 1; **HBP1C** – High Mobility Group Box Protein 1C; **OMT1** – O-Methyltransferase 1; **AAIP*** – Abscisic Acid-Induced Protein; **RPL35** – Ribosomal Protein L35; **PSBO** – Photosystem II Oxygen-Evolving Enhancer Protein 1; **RAFTIN1A** – RAFTIN1A Protein; **1-FEHw2** – Fructan 1-Exohydrolase w2; **AGP-L** – Arabinogalactan Protein-Like; **H32*** – Histone H3.2; **IF1A** – Initiation Factor 1A; **MIPS** – Myo-Inositol-1-Phosphate Synthase.

Genes marked with an asterisk (*) are wheat DEGs indicated by their name in the UniProt database.
