# Supplementary material for: Deciphering drought-response in wheat (Triticum aestivum): physiological, biochemical, and transcriptomic insights into tolerant and sensitive cultivars under dehydration shock
Source: Front Plant Sci. 2025 Oct 27;16:1649378. doi: 10.3389/fpls.2025.1649378 (PMC12598786; doi:10.3389/fpls.2025.1649378)
Supplement: Supplementary file 14 [file Table4.docx]

**Supplementary Table S4. List of cultivars used.**

|  | **Cultivar** | **The Source of the Seeds** |
| --- | --- | --- |
| 1 | Atay 85 | Transitional Zone Agricultural Research Institute-Eskişehir |
| 2 | Altay | Transitional Zone Agricultural Research Institute-Eskişehir |
| 3 | Bayraktar 2000 | Field Crops Research Institute-Ankara |
| 4 | Demir 2000 | Field Crops Research Institute-Ankara |
| 5 | Gerek 79 | Transitional Zone Agricultural Research Institute-Eskişehir |
| 6 | Harmankaya | Transitional Zone Agricultural Research Institute-Eskişehir |
| 7 | Kıraç | Transitional Zone Agricultural Research Institute-Eskişehir |
| 8 | Kırgız | Transitional Zone Agricultural Research Institute-Eskişehir |
| 9 | Müfitbey | Transitional Zone Agricultural Research Institute-Eskişehir |
| 10 | Sultan | Transitional Zone Agricultural Research Institute-Eskişehir |
| 11 | Tosunbey | Field Crops Research Institute-Ankara |
| 12 | Yıldız | Transitional Zone Agricultural Research Institute-Eskişehir |
