# Supplementary material for: Deciphering drought-response in wheat (Triticum aestivum): physiological, biochemical, and transcriptomic insights into tolerant and sensitive cultivars under dehydration shock
Source: Front Plant Sci. 2025 Oct 27;16:1649378. doi: 10.3389/fpls.2025.1649378 (PMC12598786; doi:10.3389/fpls.2025.1649378)
Supplement: Supplementary file 15 [file Table5.docx]

**Supplementary Table S5.** List of primers and their sequences used in the qRT-PCR experiments.

| **Primer Name** | **ProtID** |  | **Tm** | **GC(%)** | **PRIMER SEQ (5'-3')** | **Amplicon** |
| --- | --- | --- | --- | --- | --- | --- |
| **20F** | **>192270** | Metallo-beta-lactamase domain-containing protein | 55.31 | 50 | GTAACCTGATACCATGCCTC | 129 |
| **20R** |  |  | 54.74 | 40 | GCAGACCGTTTTACAAAGTT |  |
|  |  |  |  |  |  |  |
| **21F** | **>138222** | Ferritin (ferric iron binding; | 55.41 | 52 | CATTCTCCTGGATGACGTG | 103 |
| **21R** |  |  | 54.98 | 45 | GTTCTTCTTGATCTCGTCGA |  |
| **29F** | **>179580** | Hsp40/DnaJ-like protein | 55.02 | 45 | ACCAAAGCATTCCTCCTTAG | 119 |
| **29R** |  |  | 54.89 | 45 | TGAACCGAAGCCTATTACAG |  |
| **31F** | **>100370** | Serine/threonine protein phosphatase 2A, | 54.91 | 50 | CTAGTAGTAGAAGCACGACG | 128 |
| **31R** |  |  | 55.54 | 45 | TAAGAATACAGACTGGCCCA |  |
| **34F** | **>68053** | Protein GIGANTEA, F-box protein FBW2 | 55 | 40 | TCAACTGCGCTAATAACACT | 132 |
| **34R** |  |  | 54.86 | 45 | GCTTTCCCTTCTTGACATTG |  |
| **44F** | **>179015** | F-Box Protein, FBW2, | 55 | 45 | GAAAATCAGTCTTTGCCGAG | 105 |
| **44R** |  |  | 54.8 | 45 | AATCAAGTCCAGTAGATGCC |  |
| **45F** | **>176215** | MAPK18) | 54.8 | 45 | CACCCAAAACCGAGTAAAAG | 117 |
| **45R** |  |  | 55 | 45 | CGCGGTTTGTAATAGGAGTA |  |
| **46F** | **>172630** | Polyadenylate-binding protein RBP45B, | 54.36 | 40 | TGAAGTGCATGTCCTCAATA | 125 |
| **46R** |  |  | 54.26 | 45 | GTCTGACCAGCATTAGAGAT |  |
| **2R** | 116631 | Probable pectinesterase/pectinesterase inhibitor 42 | 55.34 | 45 | TGGACAAGATCAAGGAGAAG | 104 |
| **2R** | 116631 |  | 54.25 | 45 | ATTATTCTGCAGAGGTGTCC |  |
|  |  |  |  |  |  |  |
| **3R** | 98591 | Zinc finger CCCH domain-containing protein 36 | 55.60 | 61. | GAGAGCAAGGACCAGACC | 126 |
| **3R** | 98591 |  | 55.48 | 52.6 | GGATTCCTTGGTGTACTGC |  |
| **4R** | 98568 | Metacaspase -5 | 54.56 | 50. | TCACCAGGGATCACTAGACT | 137 |
| **4R** | 98568 |  | 55.24 | 50. | AGACACTGAGCAGCAGAGTT |  |
| **5R** | 90899 | Arogenate dehydratase 5 | 54.55 | 40 | ATGCAGCATGCTAGAACATA | 109 |
| **5R** | 90899 |  | 55.18 | 45 | AAGAATCTGAGTCATGTGGC |  |
| **11R** | 116906 | Extensin-like protein | 55.03 | 40 | AACCAGGGAAAACACATCTT | 115 |
| **11R** | 116906 |  | 54.94 | 40 | GGCAACAACAACAACAACTA |  |
| **18R** | 98579 | Germin-like protein 9-1 | 55.06 | 50 | CACCAGGGATCACTAGACTA | 102 |
| **18R** | 98579 |  | 54.96 | 40 | TGTCCGGAAATCATGAAACT |  |
